# Dynamic gain driven mode-locking in GHz fiber laser

**DOI:** 10.1038/s41377-024-01613-z

**Published:** 2024-09-20

**Authors:** Xuewen Chen, Wei Lin, Xu Hu, Wenlong Wang, Zhaoheng Liang, Lin Ling, Yang Yang, Yuankai Guo, Tao Liu, Dongdan Chen, Xiaoming Wei, Zhongmin Yang

**Affiliations:** 1 School of Physics and Optoelectronics; State Key Laboratory of Luminescent Materials and Devices; Guangdong Engineering Technology Research and Development Center of Special Optical Fiber Materials and Devices; Guangdong Provincial Key Laboratory of Fiber Laser Materials and Applied Techniques, South China University of Technology, Guangzhou, China; 2 Research Institute of Future Technology, South China Normal University, Guangzhou, Guangdong China

**Keywords:** Mode-locked lasers, Solitons

## Abstract

Ultrafast lasers have become powerful tools in various fields, and increasing their fundamental repetition rates to the gigahertz (GHz) level holds great potential for frontier scientific and industrial applications. Among various schemes, passive mode-locking in ultrashort-cavity fiber laser is promising for generating GHz ultrashort pulses (typically solitons), for its simplicity and robustness. However, its pulse energy is far lower than the critical value of the existing theory, leading to open questions on the mode-locking mechanism of GHz fiber lasers. Here, we study the passive mode-locking in GHz fiber lasers by exploring dynamic gain depletion and recovery (GDR) effect, and establish a theoretical model for comprehensively understanding its low-threshold mode-locking mechanism with multi-GHz fundamental repetition rates. Specifically, the GDR effect yields an effective interaction force and thereby binds multi-GHz solitons to form a counterpart of soliton crystals. It is found that the resulting collective behavior of the solitons effectively reduces the saturation energy of the gain fiber and permits orders of magnitude lower pulse energy for continuous-wave mode-locking (CWML). A new concept of quasi-single soliton defined in a strongly correlated length is also proposed to gain insight into the dynamics of soliton assembling, which enables the crossover from the present mode-locking theory to the existing one. Specifically, two distinguishing dynamics of Q-switched mode-locking that respectively exhibit rectangular- and Gaussian-shape envelopes are theoretically indicated and experimentally verified in the mode-locked GHz fiber laser through the measurements using both the standard real-time oscilloscope and emerging time-lens magnification. Based on the proposed criterion of CWML, we finally implement a GDR-mediated mode-locked fiber laser with an unprecedentedly high fundamental repetition rate of up to 21 GHz and a signal-to-noise ratio of 85.9 dB.

## Abstract (web summary)

This work focuses on the study of dynamic gain driven mode-locking in GHz fiber lasers and finally realizes stable mode-locking with a high fundamental repetition rate of 21 GHz.

## Graphical abstract

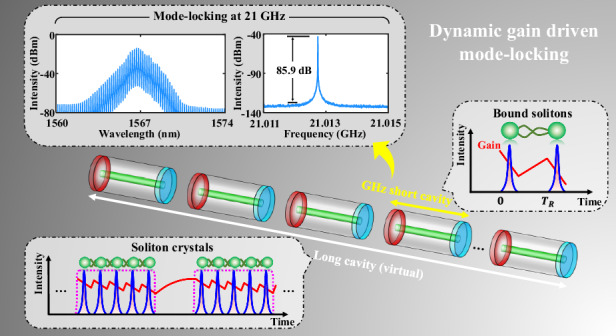

## Introduction

Ultrafast lasers with unique advantages in generating ultrashort pulses have drawn widespread interest in scientific and industrial areas, such as material processing^1^, bioimagining^2^, laser metrology^3^, and spectroscopy^4,5^. Despite the intensive studies on ultrafast lasers with kHz-MHz repetition rates, there exist strong motivations to explore these with multi-GHz fundamental repetition rates for frontier applications, like astronomical observations^6,7^, photonic microwave generation^8–10^, radar^11^, and coherent optical communications^12–16^. Driven by these crucial applications, semiconductor microring lasers^17–19^ and microresonator-filtered fiber lasers^20,21^, as well as ultrashort pulses generated in microresonators^22,23^, have been intensively investigated. Alternatively, passive mode-locking^24–27^, as a mainstay technology for generating dissipative solitons that support femtosecond pulses, is particularly promising for forming solitons with GHz fundamental repetition rates (i.e., GHz solitons) in ultrashort solid-state cavities^28,29^, although there still exist open theoretical and technical questions for mode-locked fiber lasers with fundamental repetition rates of >10 GHz^30–34^—but highly desired for their simplicity and robustness.

The pioneer theory of passive mode-locking has been investigated by exploring the master equations, and the generation of solitons using fast and slow saturable absorbers was accordingly interpreted^35–37^. This theoretical framework was further improved to establish the criterion of continuous-wave mode-locking (CWML) against Q-switched mode-locking (QSML)^38–40^, which has successfully been applied for passive mode-locking with slow gain relaxation time (${T}_{G}$). For laser materials with fast gain relaxation time close to the roundtrip time (${T}_{R}$) of laser cavities, i.e., ${T}_{G}/{T}_{R}\to 1$, these theories fail to fully understand the shaping mechanism of solitons. In this regard, the coherent master equation has subsequently been proposed to study the soliton dynamics of passive mode-locking with retaining light-matter coherence effects^41^, which is suitable for laser materials with fast gain relaxation time (typically a few nanoseconds, like semiconductors). The existing theoretical frameworks, however, encounter problems in studying passive mode-locking with complex gain dynamics, e.g., gain varying in multiple time scales. Particularly, when operating at multi-GHz fundamental repetition rates, the passively mode-locked fiber lasers face the challenge of realizing the balance between multiple effects as they are largely weakened^27,42^, and prior experimental implementations exhibit far lower pulse energy that violates the existing criteria^39^ (see Supplementary Note 1).

In this paper, we establish a new theoretical model for passively mode-locked GHz fiber lasers by exploring dynamic gain depletion and recovery (GDR) effect in dual-time scales. Specifically, it is found that the GDR-mediated collective behavior of the solitons can effectively reduce the gain saturation energy for stable mode-locking, such that the pulse energy predicted by the new criterion of CWML is in good agreement with those experimental results, which is, as a matter of fact, orders of magnitude lower than that of the existing theory. To this end, a concept of quasi-single solitons (QSSs) with different strongly-correlated (SC) lengths is also proposed for conforming the present model with the existing theory. Distinguishing dynamic landscapes in both numerical simulations and real-time experimental measurements are observed with different SC lengths, identifying the complexity and versatility of the mode-locked GHz fiber laser. We finally implement a passively mode-locked fiber laser with a fundamental repetition rate of 21 GHz—the highest one so far, to the best of our knowledge.

## Results

**Fig. 1:**
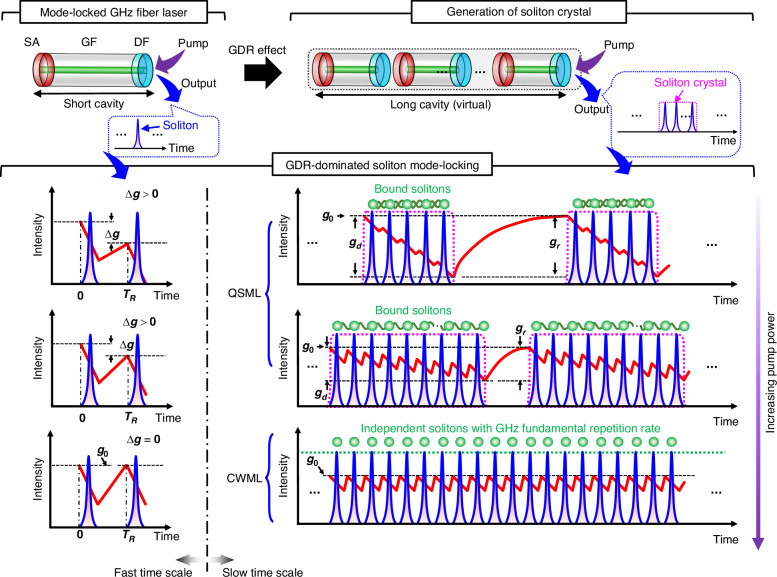
Dynamic gain driven mode-locking mechanism of the GHz fiber laser. The short cavity of the mode-locked fiber laser generates solitons with a GHz-level fundamental repetition rate (top left corner). SA, saturable absorber. GF, gain fiber. DF, dielectric film. Soliton crystals are formed from the soliton assembling process that is dominated by the gain depletion and recovery (GDR) effect, in which the laser cavity can virtually be regarded as a long one that is a cascade of multiple short cavities (top right corner). A soliton crystal contains multiple solitons separated by the roundtrip time of the short cavity. The GDR-dominated soliton assembling dynamics are individually illustrated below the short and virtual long cavities. The left side shows the dynamic gain (red curve) over a roundtrip time of the soliton (i.e., the fast time scale) at different mode-locking states, i.e., from Q-switched mode-locking (QSML) to continuous-wave mode-locking (CWML). Here, $\Delta g$ represents the gain variation over a roundtrip time. ${g}_{0}$ is the initial gain. When $\Delta g > 0$, the solitons are impacted by the dynamic gain and bound with each other. When $\Delta g=0$, the gain depletion is balanced by the gain recovery, such that the solitons become independent and unbound with each other. The right side illustrates the dynamics of soliton crystals and the corresponding dynamic gain in the slow time scale. For QSML, the gain (red curve) continuously depletes over the soliton crystal and recovers before the next soliton crystal (top and middle panels). By increasing the pump power, the length of the soliton crystal is prolonged, and the amounts of gain depletion (${g}_{d}$) and gain recovery (${g}_{r}$) between soliton crystals continuously decrease until entering the state of CWML that the depleted gain can immediately recover between solitons

Figure 1 conceptionally describes the dynamic gain driven mode-locking mechanism of the GHz fiber laser. The GHz fiber laser cavity has a Fabry-Pérot configuration (top left corner of Fig. 1) that mainly consists of a saturable absorber (SA), a gain fiber (GF), and a dielectric film (DF). The GF is pumped by a single-mode laser diode (not shown) and the soliton signal is extracted by the DF. The formation dynamics of solitons (blue) with GHz-level fundamental repetition rate (${f}_{R}$) is dominated by the GDR effect (red), through which the solitons are dynamically assembled from QSML to CWML with increasing pump power, as presented by the bottom right panels of Fig. 1.

To understand the underlying mechanism of soliton assembling, we here introduce a concept of soliton crystal that can precisely describe the GHz soliton dynamics before successful CWML, i.e., QSML at insufficient pump power. A soliton crystal consists of multiple solitons separated by the roundtrip time of the short cavity, i.e., solitons from multiple roundtrips. This is in sharp contrast to those soliton clusters involving solitons that coexist in the same roundtrip of the laser cavity that is orders of magnitude longer than the current case^43–45^, i.e., solitons from a single roundtrip. Without loss of generality, a long cavity can be virtually constructed as a cascade of multiple short cavities (top right corner of Fig. 1), such that the soliton crystal defined here contains multiple bound solitons that coexist in the same virtual long cavity, consistent with the classical definition. As a result, the dynamics of soliton assembling in the GHz fiber laser exhibit dual-time-scale features that manifest soliton behaviors in both single and multiple roundtrips of the short cavity, corresponding to the fast and slow time scales, respectively.

Here, the dynamic gain behaves in dual-time scales over the generation of solitons. In the fast time scale, GHz solitons are located close to each other, such that the gain recovery is unable to compensate for the gain depletion in forming individual solitons, as the gain is insufficient at a relatively low pump power. In general, each roundtrip pass of the soliton depletes the gain, leading to a gain variation of $\Delta g \,>\, 0$, which decreases with increasing pump power (bottom left panels of Fig. 1). The generated solitons are tightly bound through the GDR effect and finally assembled as soliton crystals (see Supplementary Note 2), i.e., presented as a soliton train modulated with rectangular-shape envelopes (pink dotted curve). The gain is continuously depleted for forming consecutive solitons within the soliton crystal, and afterwards recovers to its initial value (${g}_{0}$) before the arrival of the next soliton crystal. With increasing pump power, the rectangular-shape envelope is prolonged, and the gain variation $\Delta g$ between solitons, as well as the gain depletion (${g}_{d}$) and gain recovery (${g}_{r}$) between soliton crystals, is decreased.

The mode-locked GHz fiber laser finally transits from the QSML to CWML state when the energy of the soliton crystal conforms to the criterion of CWML. In the state of CWML, the gain variation between individual solitons is balanced, i.e., $\Delta g=0$, such that the individual solitons are presented as a continuous GHz mode-locked soliton train. More details about the gain dynamics are provided in Supplementary Note 2.1.

### Theory of mode-locking in the GHz fiber laser

#### Gain dynamics

We first theoretically investigate the gain dynamics involved in the generation of stable solitons with GHz fundamental repetition rates. The light emission of the gain fiber can be treated as a quasi-four-level system, e.g., using Er^3+^/Yb^3+^ co-doped fiber in this work. The lifetime ${T}_{G}$ of upper lasing level (i.e., gain relaxation time) in ^*4*^*I*_*13/2*_*-*^*4*^*I*_*15/2*_ translation is in a range of 1-10 ms^46^, and the rate equation of gain ($g$) can be described as

1 $$\begin{array}{c}\displaystyle\frac{{dg}}{{dt}}=-\displaystyle\frac{g-{\Lambda }_{0}}{{\tau }_{e}}-\frac{P}{{E}_{G}}g\\ \displaystyle{\rm{with}}\,{\tau }_{e}=\frac{{T}_{G}}{1+\displaystyle\frac{\varGamma {\sigma }_{a\left(p\right)}{P}_{p}{T}_{G}}{h{\upsilon }_{p}A}}\end{array}$$

where ${\tau }_{e}$, ${E}_{G}$, and ${\Lambda }_{0}$ is the effective upper-level lifetime, gain saturation energy, and small-signal gain coefficient, respectively^47^. $P$ is the instantaneous pulse power. $\varGamma$ and $A$ are the overlapping factor and effective mode area of the gain fiber, respectively. ${\sigma }_{a\left(p\right)}$ and ${\upsilon }_{p}$ are the absorption cross-section and frequency of the pump, respectively. ${P}_{p}$ is the pump power.

**Fig. 2:**
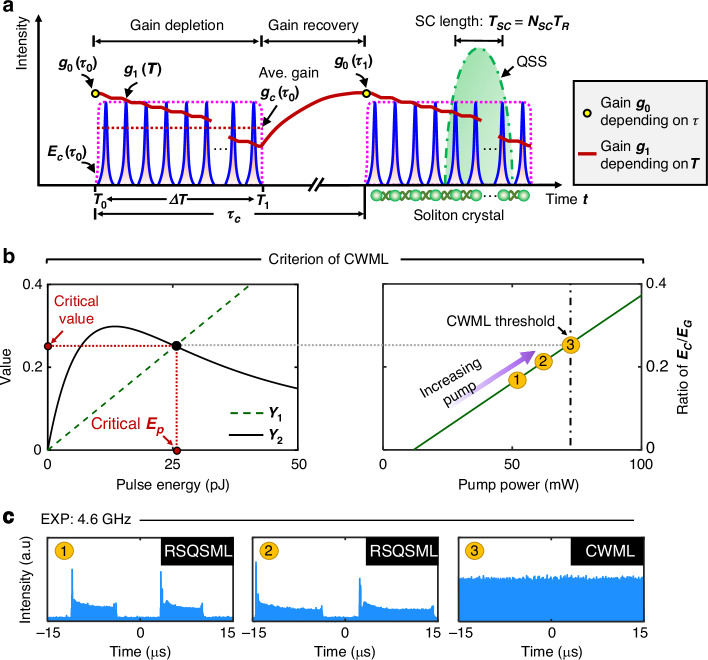
Criterion of CWML. **a** Gain dynamics in the dual-time scales. In the state of QSML, the gain is depleted and subsequently recovers between soliton crystals. The GHz solitons in the soliton crystal are bound through the GDR effect in the fast time scale. The quasi-single soliton (QSS) formed in the strongly-correlated (SC) length ${T}_{{sc}}$ is proposed to characterize the collective behavior of the soliton assembling. ${g}_{0}$, the gain depending on the slow time $\tau$. ${g}_{1}$, the gain depending on the fast time $T$. ${g}_{c}$, the average gain over the soliton crystal in the slow time scale. ${\tau }_{c}$, the period of soliton crystals. ${T}_{0}$ and ${T}_{1}$ are the start and end time of the soliton crystal, respectively. Ave., average. ${N}_{{sc}}$, the number of solitons in the SC length. ${T}_{R}$, the roundtrip time of the GHz solitons. **b** Criterion of CWML for stable mode-locking. In the left panel, the green dash and black solid curves represent the values of *Y*_1_ and *Y*_2_ as a function of the pulse energy, respectively, wherein the cross point (black dot) corresponds to the critical pulse energy for CWML, assuming ${q}_{0}=\bar{{g}_{0}}L$. The right panel shows the ratio of ${E}_{c}/{E}_{G}$ as a function of the pump power, wherein the black dash-dotted line indicates the threshold of CWML. **c** Experimental evolutions of a mode-locked fiber laser operating with a fundamental repetition rate of 4.6 GHz, i.e., evolving from rectangular-shape QSML (RSQSML) to CWML with increasing pump power

Before stable mode-locking at insufficient pump power, the solitons in the form of soliton crystals can tailor the gain. Figure 2a depicts the full picture of the gain dynamics in the dual-time scales, including both soliton-to-soliton (i.e., the fast time scale) and crystal-to-crystal (i.e., the slow time scale). To this end, a multiscale model^43^ with dual-time variables is introduced, i.e., a slow time $\tau$ accounting for the variations along crystal-to-crystal and coinciding with the physical time $t$, and a fast time $T=t/\eta$ ($\eta \ll 1$) accounting for the variations in soliton-to-soliton. By this method, the dynamic gain can be expanded as $g={g}_{0}+\eta {g}_{1}$, where ${g}_{0}$ and ${g}_{1}$ depends on the slow and fast time variables, respectively. The effective gain depletion between the solitons can be quantified by $\Delta g$, which equivalently characterizes the gain variation due to the GDR effect in the fast time scale. The gain continuously depletes over the soliton crystal and completely recovers in the time slot without solitons. The neighboring solitons are bound with each other by the GDR effect and format QSSs (green dash-dotted curve of Fig. 2a) in the SC length ${T}_{{sc}}$ (to be discussed).

Here, we derive the main theory of the gain dynamics, and the complete derivation of the equations is provided in Supplementary Note 2. In the slow time scale $\tau$, the rate equation of the gain (${g}_{0}$) is given as

2 $$\begin{array}{c}\displaystyle\frac{d{g}_{0}\left(\tau \right)}{d\tau }=-\displaystyle\frac{{g}_{0}\left(\tau \right)-{\Lambda }_{0}}{{\tau }_{e}}-\frac{{g}_{0}\left(\tau \right)\left\langle P\right\rangle }{{E}_{G}}\\ {\text{with}}\,\left\langle P\right\rangle =\displaystyle\frac{{E}_{c}\left(\tau \right)}{{\tau }_{c}}=\frac{1}{{\tau }_{c}}{\int }_{{T}_{0}}^{{T}_{1}}P\left(\tau ,T\right){dT}\end{array}$$

in which $\left\langle P\right\rangle$ is the average power over the period of the soliton crystal. ${E}_{c}\left(\tau \right)$ is the energy of the soliton crystal. $P\left(\tau ,T\right)$ is the instantaneous power of the soliton. ${\tau }_{c}$ is the period of the soliton crystal. ${T}_{0}$ and ${T}_{1}$ are the start and end times of the soliton crystal, respectively.

In the fast time scale $T$, the rate equation of the gain (${g}_{1}$) is provided as

3 $$\frac{d{g}_{1}}{{dT}}=\frac{{g}_{0}\left(\tau \right)\left\langle P\right\rangle }{{E}_{G}}-\frac{{g}_{0}\left(\tau \right)P\left(\tau ,T\right)}{{E}_{G}}$$

which describes the fast gain dynamics between the solitons. To collectively understand the gain dynamics among both individual solitons and soliton crystals, we consider the depleted gain $g\left(\tau ,t\right)$ over the soliton crystal in the unsaturated condition, and arrive at

4 $$g\left(\tau ,t\right)={g}_{0}\left(\tau \right)\exp \left(-\frac{1}{{E}_{G}}{\int }_{{\tau }_{0}}^{t}P\left({t}^{{\prime} }\right)d{t}^{{\prime} }\right)$$

Thus, the average gain over the soliton crystal becomes

5 $${g}_{c}\left(\tau \right)={\int }_{{\!\tau }_{0}}^{{{\tau }_{0}+\tau }_{c}}g\left(\tau ,t\right)f\left(t\right){dt}={g}_{0}\left(\tau \right)\frac{1-\exp \left(-{E}_{c}\left(\tau \right)/{E}_{G}\right)}{{E}_{c}\left(\tau \right)/{E}_{G}}$$

where the normalized function $f\left(t\right)$ describes the envelope of the soliton crystal that is expressed as$\,{\int }_{0}^{{\tau }_{c}}f\left(t\right){dt}=1$.

#### Mode-locking through the GDR effect

In contrast to the existing theoretical models of passive mode-locking^35–37^ as well as those improved ones^38,39^, here we explore the mode-locking mechanism of GHz fiber lasers that is dominated by the gain dynamics with dual-time scales. Instead of focusing on individual solitons, we navigate the dynamic behavior of the soliton crystal. Then, the coupled rate equations that govern the stability of the soliton crystal can be written as

6a $$\frac{d{E}_{c}\left(\tau \right)}{d\tau }=\left[{\frac{2L}{{T}_{R}}}{g}_{c}\left(\tau \right)-\frac{{q}_{l}}{{T}_{R}}-\frac{q}{{T}_{R}}\right]{E}_{c}\left(\tau \right)$$

6b $$\begin{array}{c}\begin{array}{c}\displaystyle\frac{d{g}_{0}(\tau )}{d\tau }=-\displaystyle\frac{{g}_{0}(\tau )-{\Lambda }_{0}}{{\tau }_{e}}-\frac{{g}_{0}(\tau ){E}_{c}\left(\tau \right)}{{E}_{G}{\tau }_{c}}\\ {\rm{with}}\,q=\displaystyle\frac{{q}_{0}\left[1-\exp \left(-{E}_{c}\left(\tau \right){T}_{R}/{E}_{a}\Delta T\right)\right]}{{E}_{c}\left(\tau \right){T}_{R}/{E}_{a}\Delta T}\end{array}\end{array}$$

where $q$, ${q}_{l}$, ${q}_{0}$, and ${E}_{a}$ are the saturable absorption, linear loss, modulation depth, and saturable energy of the saturable absorber, respectively. *L* is the total length of laser cavity. $\Delta T$ is the duration of the soliton crystal. According to Eq. (6), the ordinary differential equations of the perturbations for the soliton crystal energy ${E}_{c}$ and gain ${g}_{0}$ can be expressed in the vector form, i.e.,

7 $$\begin{array}{c}{T}_{R}\displaystyle\frac{d}{d\tau }\left(\begin{array}{c}{\delta E}_{c}\\ \delta {g}_{0}\end{array}\right)={M}_{J}\left(\begin{array}{c}{\delta E}_{c}\\ \delta {g}_{0}\end{array}\right)\\ {\rm{with}}\,{M}_{J}=\left(\begin{array}{cc}-{q}_{l}-{q}_{0}{e}^{-\bar{{E}_{c}}{T}_{R}/{E}_{a}\Delta T}+2\bar{{g}_{0}}L{e}^{-\bar{{E}_{c}}/{E}_{G}} & 2{E}_{G}L\left(1-{e}^{-\bar{{E}_{c}}/{E}_{G}}\right)\\ \displaystyle-\frac{{T}_{R}\bar{{g}_{0}}}{{E}_{G}{\tau }_{c}} & \displaystyle-\left(\frac{{T}_{R}}{{\tau }_{e}}\,+\,\frac{{T}_{R}\bar{{E}_{c}}}{{E}_{G}{\tau }_{c}}\right)\end{array}\right)\end{array}$$

where $\left(\bar{{E}_{c}},\,\bar{{g}_{0}}\,\right)$ represents the fixed point of Eq. (6). Following a standard procedure of linear stability analysis, we derive a new criterion of CWML for passively mode-locked fiber lasers operating with GHz fundamental repetition rates, i.e.,

8 $$\mathop{\mathrm{lim}}\limits_{\Delta T\to {\tau }_{c}}\frac{\bar{{E}_{c}}}{{E}_{G}}=\mathop{\underbrace{\frac{{E}_{p}{f}_{R}}{{E}_{G}{f}_{c}}}}\limits_{{Y}_{1}} > \mathop{\underbrace{\frac{{q}_{0}}{\bar{{g}_{0}}L}\left(\frac{\left[1-\exp \left(-{E}_{p}/{E}_{a}\right)\right]}{{E}_{p}/{E}_{a}}-{e}^{-{E}_{p}/{E}_{a}}\right)}}\limits_{{Y}_{2}}$$

For strongly saturated saturable absorbers, i.e., ${E}_{p}/{E}_{a}\gg 1$, it gives rise to a simplified formalism, i.e.,

9 $$\frac{\overline{{E}_{c}}}{{E}_{G}} > \frac{{q}_{0}}{\overline{{g}_{0}}L}\frac{{E}_{a}}{{E}_{p}}\,\mathop{\Longrightarrow }\limits^{\varDelta T\to {\tau }_{c}}\,{E}_{p}^{2} \,>\, \frac{{q}_{0}{f}_{c}}{\overline{{g}_{0}}L{f}_{R}}{E}_{a}{E}_{G}$$

where ${f}_{c}$ is the repetition rate of soliton crystals. It is noticed that Eq. (9) has a similar formalism as that of the classical standard criterion^39^, i.e., ${E}_{p}^{2} \,>\, {q}_{0}{E}_{a}{E}_{G}$. Interestingly, the dynamic gain mediated soliton assembling can reduce the pulse energy requirement for CWML. To understand the collective behavior of soliton assembling, we define the number (${N}_{{SC}}$) of bound solitons in a QSS as ${N}_{{SC}}=\sqrt{\frac{\bar{{g}_{0}}L{f}_{R}}{{f}_{c}}}$, and rewritten Eq. (9) as

10 $${\left({N}_{{sc}}{E}_{p}\right)}^{2} \;>\; {q}_{0}{E}_{a}{E}_{G}$$

As illustrated in Fig. 2a, a single QSS comprises a number ${N}_{{SC}}$ of bound solitons, which are correlated in the SC length ${T}_{{SC}}$ (${T}_{{SC}}={N}_{{SC}}{T}_{R}$). The detailed derivation is provided in Supplementary Note 2.2. Without loss of generality, the QSS becomes the elementary unit of the virtual long cavity, which allows a better understanding of the mode-locking dynamics in GHz fiber laser (to be discussed).

Figure 2b illustrates the criterion for CWML with GHz fundamental repetition rates, i.e., Eq. (8). Intuitively, CWML can be realized at the point where the value of ${Y}_{1}$ crosses with the value of ${Y}_{2}$, leading to the critical pulse energy and the corresponding critical value. The right panel of Fig. 2b depicts the ratio of ${E}_{c}/{E}_{G}$ as a function of the pump power, wherein CWML is obtained with ${E}_{c}/{E}_{G}$ equal to the critical value (see Supplementary Note 2). Figure 2c showcases the typical evolutions of GHz soliton trains at different levels of pump power in the experiments, confirming that the mode-locking could transit from rectangular-shape QSML (RSQSML) to CWML with increasing pump power.

### Dynamics of QSML with different SC lengths

**Fig. 3:**
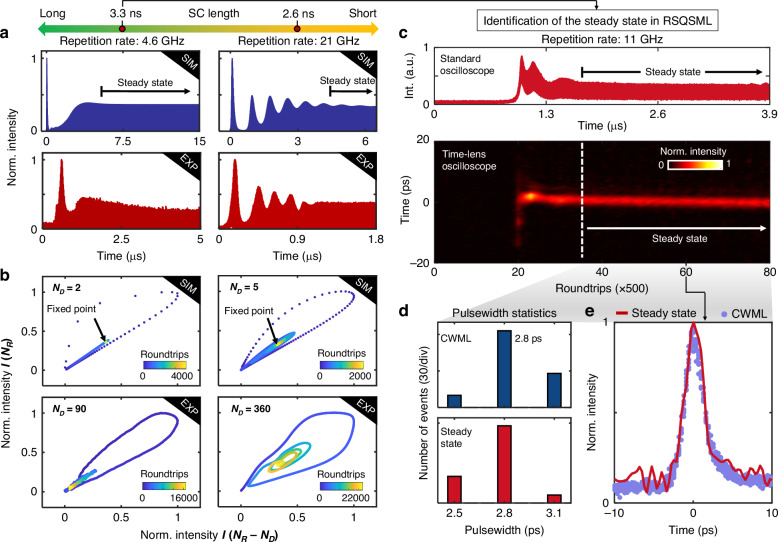
Dynamics of RSQSML with different SC lengths altered by changing the fundamental repetition rate. **a** Simulated (top) and experimental (bottom) evolutions of RSQSML with different SC lengths, i.e., 3.3 and 2.6 ns. The SC length is altered by changing the fundamental repetition rate, e.g., 4.6 and 21 GHz for the left and right columns, respectively. **b** Simulated (top) and experimental (bottom) phase space orbits with different SC lengths as that of (**a**). The results are reconstructed from the pulse signal of (**a**), which is delayed by a roundtrip number of ${N}_{D}$, i.e., 2, 5, 90, and 360, respectively. **c** Steady state of RSQSML with a SC length of 3.3 ns measured by a standard real-time oscilloscope (top) and time-lens magnification measurement system (bottom), respectively. Corresponding simulated results are given in Fig. S3 and exhibit good consistency with the dynamics of QSML in the experiment. **d** Pulsewidth statistics for CWML (top) and steady state of RSQSML (bottom), respectively. **e** Pulse waveforms for CWML (violet) and steady state of RSQSML (red), respectively

The QSML contains versatile interesting dynamics for exploring the novel mechanism of mode-locking in GHz fiber lasers. We here theoretically and experimentally investigate the state of QSML by modeling it according to the concept of the QSS. The numerical simulation of the QSML dynamics is performed by leveraging the nonlinear Schrödinger equation (NLSE), rate equations of saturable absorption, and dynamic gain of the QSS. More details are provided in Methods and Supplementary Note 2.2. Figure 3 shows the numerical and experimental results of the QSML dynamics with different ${T}_{{SC}}$. By changing the ${T}_{{SC}}$, distinctive evolutionary behaviors of QSML are observed, wherein a good agreement between the simulation and experiment justifies the validity of the QSS concept, as shown in Fig. 3a. For a relatively long ${T}_{{SC}}$, e.g., left column of Fig. 3a, the gain instantaneously depleted by the QSS has sufficient time to recover, which facilitates a timely transition to the steady state and gives rise to flat-top soliton crystals, i.e., RSQSML as shown in Fig. 2c. For a shorter ${T}_{{SC}}$, the gain recovery between the QSSs is limited, and it manifests the oscillatory evolution before the transition to the steady state (right column of Fig. 3a).

Figure 3b depicts the numerical and experimental phase space orbits with different ${T}_{{SC}}$, which are reconstructed from the pulse signal and its delay by a roundtrip number of ${N}_{D}$. The evolving trajectories end at a fixed point corresponding to the final steady state of RSQSML. In contrast to the case with longer ${T}_{{SC}}$, the phase space orbit with shorter ${T}_{{SC}}$ is vortical, resulted from the oscillatory structures before converging to the fixed point (right column of Fig. 3a). To further identify the steady state of RSQSML, the dynamics of RSQSML with ${T}_{{SC}}$ of 3.3 ns are recorded by a time-lens magnification measurement system implemented by using the space-time duality^48,49^ (see Methods and Supplementary Note 3), as shown in the bottom panel of Fig. 3c, and the corresponding measurement using a standard real-time oscilloscope is also provided in the top panel of Fig. 3c.

The pulsewidth statistics indicate that most of the solitons have a pulsewidth of ~2.8 ps in both CWML and the steady state of RSQSML (Fig. 3d). Figure 3e further shows the pulse waveforms in both cases, which are measured by the time-lens magnification measurement system. The consistent results suggest that the solitons in the steady state of RSQSML exhibit identical properties, resulting in a transient case of dynamic gain driven mode-locking. However, the solitons in the steady state of RSQSML are subjected to the GDR effect and they could not live for long. This is because ${g}_{0}$ still suffers from the gain depletion accumulated throughout the soliton crystal, but the solitons would appear again after the gain recovery that takes place in the time slot without solitons. In CWML, nevertheless, ${g}_{0}$ remains, such that the formation of the solitons is under the balance between gain and loss in the fast time scale, giving rise to the stable GHz solitons.

**Fig. 4:**
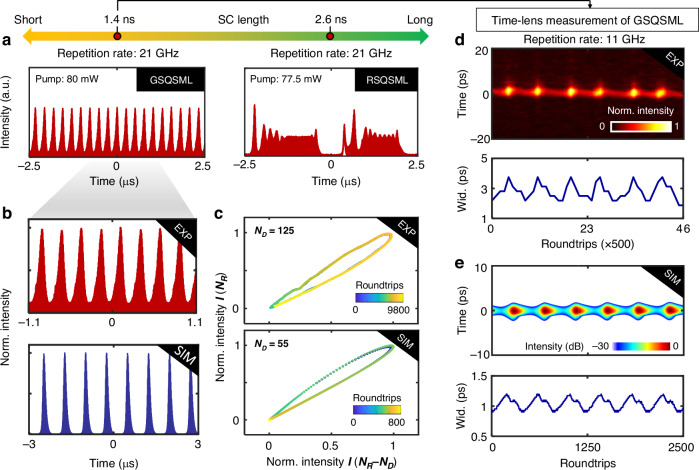
Dynamics of GSQSML with a decreased SC length altered by changing the pump power. **a** Experimental evolution of QSML with a fixed fundamental repetition rate of 21 GHz at different levels of pump power. The results are recorded by the standard real-time oscilloscope. Here, the GHz fiber laser evolves from RSQSML (right) to GSQSML (left) as the pump power increases from 77.5 mW to 80 mW. **b** Closeup of the experimental GSQSML evolution (top) and corresponding simulation (bottom). **c** Experimental (top) and simulated (bottom) phase space orbits of **b**, delayed by ${N}_{D}=$ 125 and 55, respectively. **d** Time-lens magnification measurement of GSQSML. The top panel shows the temporal evolution of the GSQSML signal, while the bottom panel illustrates the variation of its pulsewidth. Here, the SC length is 1.4 ns. Wid. is short for pulse width. **e** Simulation of GSQSML corresponding to **d**

Figure 4a illustrates other dynamics of QSML with distinguishing evolutionary features. Rather than varying the fundamental repetition rate as that of Fig. 3, here the SC length is changed by manipulating the dynamic gain with a fixed fundamental repetition rate at 21 GHz, i.e., tuning the pump power. Interestingly, a transition state of Gaussian-shape QSML (GSQSML) exists in between the RSQSML and CWML, as the pump power pump increases from 77.5 to 80 mW. This GSQSML state exhibits similar features as that of QSML with low repetition rates^40^. This is because the QSS, as the elementary unit here, behaves like a soliton as considered in the existing theory and then emulates the classical Q-switched dynamics (see Supplementary Note 2.3). To gain more information about the GSQSML dynamics, the numerical simulation is performed, and a good agreement with the experiment is obtained, as depicted in Fig. 4b, c. In contrast to that of Fig. 3b, here the evolutionary trajectories exhibit limit cycles in both the simulation and experiment, indicating that the peak power of the soliton periodically varies in the GSQSML state. To access more details of the evolving soliton, the time-lens magnification measurement is conducted for the GSQSML state with a decreased SC length (i.e., 1.4 ns in this case). The dynamic landscape of the temporal evolution is visualized (top panel of Fig. 4d) and a breathing variation of the pulsewidth is observed (bottom panel of Fig. 4d), both of which are well reproduced by the simulation (Fig. 4e) and further verify the validity of the QSS concept for understanding the QSML in the GHz fiber laser.

### Generation of stable CWML solitons with an unprecedentedly high fundamental repetition rate in a fiber laser

**Fig. 5:**
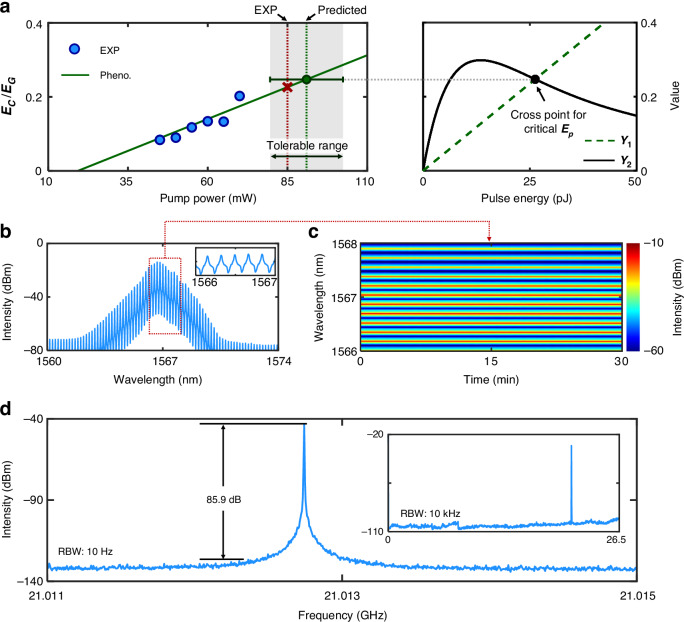
Generation of stable CWML solitons with a fundamental repetition rate of 21 GHz. **a** Criterion of generating stable 21-GHz solitons in the experiment and the corresponding phenomenological prediction. The left panel depicts the experimental result (blue dots), and phenomenological prediction (green solid line) by associating the energy ratio of ${E}_{c}/{E}_{G}$ with the critical value of ${Y}_{2}$. The red cross and green dot, respectively, indicate the experimental and theoretically-predicted thresholds of CWML with a fundamental repetition rate of 21 GHz. The theoretical study identifies that the CWML state can tolerate a moderate range of pump power, i.e., the gray area. The right panel presents the values of ${Y}_{1}$ and ${Y}_{2}$ as a function of the pulse energy for stable mode-locking. **b** Optical spectrum of the generated stable 21-GHz solitons. The inset shows the closeup of (**b**). **c** Stability measurement of the optical spectrum over 30 minutes. **d** Radio-frequency (RF) spectrum, measured with a resolution bandwidth (RBW) of 10 Hz. The inset shows the RF spectrum in a wide frequency span, i.e., from 0 to 26.5 GHz, measured with an RBW of 10 kHz

So far, we have experimentally and theoretically investigated the working principle of the mode-locked GHz fiber laser, and it is interesting to implement the generation of stable solitons with unprecedented high fundamental repetition rates for frontier applications. Figure 5a shows the experimental result and phenomenological prediction of generating stable solitons with a fundamental repetition rate of up to 21 GHz in the fiber laser. The prediction is based on the proposed criterion of Eq. (8), wherein the value of ${Y}_{2}$ at critical ${E}_{p}$ is associated with a phenomenological energy ratio of ${E}_{c}/{E}_{G}$ to derive the pump threshold of CWML. It predicts that the CWML can be realized at a pump power of around 90 mW subject to a moderate tolerable range (see Supplementary Note 4). In our implementation, at a pump power of 85 mW, the GHz fiber laser works in the CWML state, i.e., the red cross of Fig. 5a, with a clean optical spectrum (Fig. 5b) due to a small amount of net intracavity dispersion^50^. The unprecedented high fundamental repetition rate is manifested by the large longitudinal mode spacing, i.e., 0.17 nm (right inset of Fig. 5b), wherein the linewidth of the comb lines exhibits to be broad due to the limited spectral resolution of the optical spectrum analyzer (i.e., 0.02 nm). To characterize the stability of the CWML GHz fiber laser, its optical spectrum is monitored, as depicted in Fig. 5c. To analyze the radio-frequency (RF) signal of the GHz fiber laser in a narrow span, Fig. 5d illustrates the RF spectrum measured with a resolution bandwidth (RBW) of 10 Hz, which indicates a fundamental repetition rate of 21 GHz and a signal-to-noise ratio (SNR) of up to 85.9 dB. Besides, a full-span one with a larger RBW of 10 kHz is also measured and shown in the inset of Fig. 5d, exhibiting a clean RF signal.

## Discussion

To summarize, we have unveiled a dynamic gain driven mode-locking mechanism in fiber lasers with GHz fundamental repetition rates and developed a theoretical framework incorporating the gain dynamics in dual-time scales for comprehensively understanding their soliton dynamics. The dynamic gain landscape mediated by the GDR effect was investigated, and the involved soliton assembling gave rise to the formation of soliton crystals. The collective behavior of multiple solitons permitted CWML at much lower pulse energy as it effectively reduced the gain saturation energy. Based on this theoretical framework, a new criterion of CWML in GHz fiber lasers was proposed, and it well explained the reason why the pulse energy for stable mode-locking with GHz fundamental repetition rates is usually far lower than the criterion of the existing theory. Furthermore, we bridged the present theoretical framework with the existing theory by introducing the concept of the QSS. By manipulating the SC length of the QSS, distinguishing mode-locking dynamics, i.e., RSQSML and GSQSML, were explicitly reproduced. Both standard real-time oscilloscope and emerging time-lens magnification measurements were conducted, and the results are in good consistency with the numerical simulations, verifying the validity of the QSS concept. Finally, a stable mode-locked fiber laser with an unprecedentedly high fundamental repetition rate of up to 21 GHz was demonstrated with an SNR of up to 85.9 dB. Such an all-fiber soliton source could be readily integrated with a piezoelectric transducer for frequency stabilization, as we showcase in Supplementary Note 5, creating new potential for high-repetition-rate optical frequency comb, photonic microwave generation, coherent optical communication, etc. These efforts may also shed new light on passive mode-locking in other kinds of microcavity lasers with more complex gain dynamics.

## Materials and methods

### Experimental setup

The mode-locked GHz fiber lasers used in the experimental studies were configurated with Fabry-Pérot cavities that consist of a semiconductor saturable absorption mirror (SESAM), a dielectric film (DF), and a piece of homemade heavily Er^3+^/Yb^3+^ co-doped gain fiber (GF) with a net gain coefficient of 9.1 dB cm^−1^. Three mode-locked fiber lasers with different lengths of GF were constructed, i.e., about 21.4, 8.9, and 4.7 mm for fundamental repetition rates of 4.6, 11, and 21 GHz, respectively. The SESAM used for mode-locking has a saturation fluence of 15 μJ·cm^−2^ (Batop GmbH SAM-1550-10-5ps for the fundamental repetition rate of 4.6 GHz, while SAM-1550-7-10ps for both 11 and 21 GHz). The fiber-type DF has a transmittance of 99.5% at 974 nm (i.e., the pump wavelength) and a high reflection of ~99% at 1530 to 1570 nm (i.e., the signal wavelength). The homemade GF was pumped by a 974-nm single-mode laser diode (LD) through a 974/1550-nm wavelength-division multiplexer (WDM). An isolator (ISO) was connected to the output port of the WDM to prevent the back reflection. To stabilize the fundamental repetition rate, a piezoelectric transducer was integrated with the GHz fiber laser cavity to implement the phase-locked loop (Fig. S9). More details are provided in Supplementary Note 5.

### Data acquisition

The output power of the mode-locked GHz fiber laser was monitored by a photodiode-based power meter (Thorlabs S122C), while the optical spectrum was analyzed by an optical spectrum analyzer (YOKOGAWA AQ6370D). The optical pulses were converted to electric signals via a high-speed photodetector (New Focus 1414, 25 GHz bandwidth), and recorded by a standard real-time oscilloscope (Keysight DSOV204A, 20 GHz bandwidth). The radio-frequency (RF) performance of the mode-locked GHz fiber laser, like phase noise and timing jitter, was analyzed by an RF spectrum analyzer (Rohde & Schwarz FSWP26, 26.5 GHz bandwidth). The temporal waveform of the mode-locked pulses was measured by an autocorrelator (APE Pulsecheck USB 50, 50 ps scan range) and a homebuilt time-lens magnification measurement system (see Supplementary Note 3).

### Modeling of the quasi-single soliton (QSS)

The soliton assembling dominated by the dynamic gain exhibits collective behavior of the QSS, in which way it fits the master equations akin to that of a classical lumped model, i.e.,

11a $$\frac{\partial {u}_{i}}{\partial z}=\left(-i\frac{{\beta }_{2}}{2}+\frac{g\left(z,{\tau }_{i}\right)}{{\Omega }_{g}^{2}}\right)\frac{{\partial }^{2}{u}_{i}}{\partial {T}^{2}}+i\gamma {\left|{u}_{i}\right|}^{2}{u}_{i}+g\left(z,{\tau }_{i}\right){u}_{i}$$

11b $$\frac{{dq}}{{dT}}=-\frac{q-{q}_{0}}{{\tau }_{a}}-\frac{{{|u|}}^{2}}{{E}_{a}}q$$

11c $$\frac{\partial g\left(z,\tau \right)}{\partial \tau }=-\frac{g\left(z,\tau \right)-{\Lambda }_{0}}{{\tau }_{e}}-\frac{{{||u||}}^{2}}{{E}_{G,{eff}}{T}_{{SC}}}g\left(z,\tau \right),{\rm{with}}\,{E}_{G,{eff}}=\frac{{E}_{G}}{{N}_{{SC}}^{2}}$$

where ${\beta }_{2}$, ${\Omega }_{g}$, and $\gamma$ are the second-order dispersion, gain bandwidth, and nonlinearity of gain fiber, respectively. ${\tau }_{a}$ is the relaxation time of the saturable absorber. Equation (11a–c) describe the *z*-evolving optical field ${u}_{i}$ of the QSS by leveraging the saturable absorption of the SESAM and dynamic gain of the GF. The QSS, in analogy with the existing mode-locking model, is defined in a range of [−${T}_{R}/2$, ${T}_{R}/2$] (${T}_{R}$, roundtrip time). To conform the equivalent GDR-driven effect casted by ${N}_{{sc}}$-soliton, an effective gain saturation energy ${E}_{G,{eff}}$ is introduced and it satisfies

12 $${\left({N}_{{sc}}{E}_{p}\right)}^{2} \,>\, {q}_{0}{E}_{a}{E}_{G}\,\Longrightarrow\, {E}_{p}^{2} \,>\, {q}_{0}{E}_{a}{E}_{G,{eff}}$$

In the numerical simulation, the step used in the slow time scale $\tau$ is ${T}_{{SC}}$, and we have ${\tau }_{i}=i{T}_{{SC}}$ that coincides with the strongly-correlated (SC) length of QSS. The key parameters used in the numerical simulation are provided in Table 1.

**Table 1:** Key parameters used in the numerical simulation

Parameter	Value
**Fiber parameter**	
GF dispersion (${\beta }_{2}$, ps^2^ km^−1^)	−10
GF nonlinearity ($\gamma$, W^−1^ km^−1^)	3
**SESAM and dielectric film parameter**	
Unsaturable loss	0.06 (for 4.6 GHz) 0.04 (for 11 & 21 GHz)
Modulation depth (${q}_{0}$)	0.04 (for 4.6 GHz) 0.03 (for 11 & 21 GHz)
Relaxation time (${\tau }_{a}$, ps)	5 (for 4.6 GHz) 10 (for 11 & 21 GHz)
Dispersion (fs2)	-2000 (for 4.6 GHz) 1025 (for 11 & 21 GHz)
Saturation energy (${E}_{a}$, pJ)	7.5
Output ratio	0.01
**Gain characteristics**	
Small-signal gain (${\Lambda }_{0}$, m^−1^)	150 (for 4.6 & 21 GHz) 100 (for 11 GHz)
Gain bandwidth (${\Omega }_{g}$, nm)	24
Effective upper-level lifetime (${\tau }_{e}$, μs)	14
Effective gain saturation energy (${E}_{G,{eff}}$, nJ)	2 (for 4.6 GHz) 3 (for 11 & 21 GHz)

### Time-lens magnification measurement system

A homebuilt time-lens magnification measurement system is employed to temporally magnify the GHz optical pulses from the mode-locked fiber laser. The pump beam of the time-lens magnification measurement system was provided by an all polarization-maintaining (PM) figure-9 Er-doped fiber laser with a fundamental repetition rate of ~19.6 MHz. The repetition rate of the pump source can be continuously tuned in a range of 40 kHz using an intracavity variable optical delay line (ODL), so as to realize a short-term synchronization with the signal under test (SUT). The generated pump pulse first passed through a bandpass filter at 1550 nm (13 nm bandwidth) and was then chirped by a spool of dispersion compensating fiber (DCF) with a total group delay dispersion (GDD) of -39 ps nm^−1^. Before combining with the SUT, it was amplified by an erbium-doped fiber amplifier (EDFA). As for the SUT, i.e., the GHz-repetition-rate pulses centered at ~1565 nm, it was pre-amplified to tens of mW and sent to another segment of DCF with a GDD of -19 ps nm^−1^. The ODL was placed in the signal branch, which allows for fine-tuning the relative temporal position between the pump and signal pulses. Subsequently, the pump pulse and SUT were combined by a broadband optical coupler. The phase impartation was implemented via a four-wave mixing (FWM) process within a highly nonlinear fiber (HNLF, YOFC NL-1550-Zero, 10 m length). Consequently, as the optical field after the FWM passed through a bandpass filter at 1535 nm (6 nm bandwidth), the generated idler component was extracted and further amplified by another EDFA. To eventually complete the time-lens imaging, a DCF with a GDD of -760 ps nm^−1^ was employed, and the final output was detected by a high-speed photodiode (Newport 1544-B, 12.5 GHz bandwidth) and recorded by the standard real-time oscilloscope. More details about time-lens magnification measurement system are also provided in Supplementary Note 3.

## Supplementary information

Supplementary information for Dynamic gain driven mode-locking in GHz fiber laser

## Data Availability

All data used in this study are available from the corresponding authors upon reasonable request.

## Code availability

All custom codes used in this study are available from the corresponding authors upon reasonable request.
