# Supplementary material for: Dynamic gain driven mode-locking in GHz fiber laser
Source: Light Sci Appl. 2024 Sep 20;13:265. doi: 10.1038/s41377-024-01613-z (PMC11413297; doi:10.1038/s41377-024-01613-z)
Supplement: Supplementary file 1 — Supplementary information for Dynamic gain driven mode-locking in GHz fiber laser [file 41377_2024_1613_MOESM1_ESM.docx]

Supplementary Information for:

**Dynamic gain driven mode-locking in GHz fiber laser**

Xuewen Chen^1,†^, Wei Lin^1,†^, Xu Hu^1,†^, Wenlong Wang^1^, Zhaoheng Liang^1^, Lin Ling^1^, Yang Yang^1^, Yuankai Guo^1^, Tao Liu^1^, Dongdan Chen^1^, Xiaoming Wei^1,*^, and Zhongmin Yang^1,2,*^

^1^School of Physics and Optoelectronics; State Key Laboratory of Luminescent Materials and Devices; Guangdong Engineering Technology Research and Development Center of Special Optical Fiber Materials and Devices; Guangdong Provincial Key Laboratory of Fiber Laser Materials and Applied Techniques, South China University of Technology, Guangzhou 510640, China.

^2^Research Institute of Future Technology, South China Normal University, Guangzhou, Guangdong 510006, China

^†^These authors contributed equally: Xuewen Chen, Wei Lin, Xu Hu.

^*^Correspondence should be addressed to X.M.W. (xmwei@scut.edu.cn) or Z.M.Y. ([yangzm@scut.edu](mailto:yangzm@scut.edu).cn).

Supplementary Note 1: Summary of passively mode-locked fiber lasers with GHz fundamental repetition rates

We summarize the previous works of GHz-fundamental-repetition-rate passively mode-locked fiber lasers at different wavelengths, i.e., **Table S1**, wherein the critical energy of continuous-wave mode-locking (CWML) in the experiment is compared with the theoretical prediction of the existing mode-locking theory.

**Table S1 | Passively mode-locked fiber lasers with GHz fundamental repetition rates**

| Fiber laser | Fundamental repetition rate | Center wavelength | Saturable absorber | Pulse energy in experiment | Pulse energy of existing theory |
| --- | --- | --- | --- | --- | --- |
| Yb-fiber laser^1^ | 3 GHz | 1026 nm | SESAM | >200 pJ | 4 nJ |
| Yb-fiber laser^2^ | 5 GHz | 1059 nm | SESAM | >20 pJ | 1.5 nJ |
| Yb-fiber laser^3^ | 3 GHz | 1042 nm | SESAM | >36.4 pJ | 1.5 nJ |
| Yb-fiber laser^4^ | 7 GHz | 1050 nm | SESAM | ~8.6 pJ | 1.5 nJ |
| Er-fiber laser^5^ | 1 GHz | 1573 nm | SESAM | 283 pJ | 1.5 nJ |
| Er-fiber laser^6^ | 3 GHz | 1555 nm | SESAM | 33 pJ | 2.8 nJ |
| Er-fiber laser^7^ | 1 GHz | 1554 nm | SESAM | >4 pJ | 1.7 nJ |
| Er-fiber laser^8^ | 2.7 GHz | 1558 nm | SESAM | >10.6 pJ | 1.7 nJ |
| Er-fiber laser^9^ | 1 GHz | 1535 nm | SESAM | 18.5 pJ | 10.8 nJ |
|  | 2.2 GHz | 1534 nm |  | 16.1 pJ | 10.8 nJ |
| Er-fiber laser^10^ | 5 GHz | 1561 nm | SESAM | 16.2 pJ | 1 nJ |
| EY-fiber laser^11^ | 1 GHz | 1533 nm | SESAM | 65 pJ | 0.9 nJ |
|  | 2 GHz | 1535 nm | SESAM | 50 pJ | 0.9 nJ |
| EY-fiber laser^12^ | 4.2 GHz | 1601 nm | CNT | >15 pJ | / |
|  | 9.6 GHz | 1560 nm |  | >26 pJ | / |
|  | 19.5 GHz | 1563 nm |  | >65 pJ | / |
| EY-fiber laser  **(This work)** | 21 GHz | 1567 nm | SESAM | ~24 pJ | 1.5 nJ |
| EY-fiber laser^13^ | 12 GHz | 1535 nm | SESAM | >8 pJ | 7.5 nJ |
| EY-fiber laser^14^ | 3.2 GHz | 1563 nm | SESAM | ~68 pJ | 4 nJ |
| EY-fiber laser^15^ | 4.9 GHz | 1564 nm | SESAM | 23 pJ | 1.8 nJ |
| EY-fiber laser^16^ | 12.5 GHz | 1564 nm | SESAM | 30 pJ | 1.8 nJ |
| EY-fiber microresonator^17^ | 9.7 GHz | 1.5 μm, tunable | Graphene | 31 pJ | / |
| Tm-fiber laser^18^ | 1.6 GHz | 1960 nm | SESAM | 19.7 pJ | 4.9 nJ |
| Tm-fiber laser^19^ | 1.3 GHz | 1940 nm | SESAM | 160 pJ | 1.9 nJ |
| Tm-fiber laser^20^ | 4.3 GHz | 1968 nm | SESAM | 3.1 pJ | 3.5 nJ |
| Tm-fiber laser^21^ | 11.3 GHz | 1915 nm | SESAM | 1 pJ | 1.9 nJ |

SESAM: semiconductor saturable absorber mirror. CNT, carbon nanotube. The pulse energy of the existing theory is calculated by $E_{criterion}=\sqrt{q_{0}E_{a}E_{G}}$, where $q_{0}$ and $E_{a}$ are the modulation depth and saturation energy of the saturable absorber, respectively. $E_{G}$ is the gain saturation energy.

For both previous and present works, **Table S1** shows that most of the passively mode-locked fiber lasers with GHz fundamental repetition rates have far lower pulse energies compared with the predictions of the existing theory. Notably, compared to the GHz fiber laser mode-locked by other type of saturable absorber (e.g., Ref. [12]), the present laser mode-locked by semiconductor saturable absorber mirror (SESAM) delivers GHz solitons with a slightly higher fundamental repetition rate and a better radio-frequency (RF) SNR.

Supplementary Note 2: Mode-locking mechanism of GHz fiber lasers

In passively mode-locked GHz fiber lasers, the significant difference from these typical long-cavity fiber lasers is the increasing soliton-to-soliton interaction resulted from the gain depletion and recovery (GDR) effect. In this work, we explore the new mechanism of mode-locking that is dominated by the GDR effect for GHz fiber lasers using rare-earth (RE)-doped fibers as the gain media. With fundamental repetition rates of up to multi-GHz, there exists a non-zero differential gain $\Delta g=\left( {\delta g}_{d}-{\delta g}_{r} \right)$over the roundtrip time of the short cavity, since the time interval between the solitons is significantly short for gain recovery (**Fig. S1**). As a result, the soliton assembling dominated by the GDR effect plays a significant role in mode-locking as well as underlying dynamics. To evaluate the differential gain, we use a standard rate equation of gain $g$, i.e.,

$$\begin{aligned} \frac{dg}{dt}=-\frac{g-\Lambda_{0}}{\tau_{e}}-\frac{\left\| u \right\|}{E_{G}T_{R}}g,\#\left( S1 \right) \end{aligned}$$

where $\Lambda_{0}$ and $E_{G}$ are the small-signal gain coefficient and gain saturation energy, respectively. $\tau_{e}$ is the effective upper-level lifetime^22^. $\left\| u \right\|$ represents the single pulse energy. The dynamic gain can be modeled with a gain depletion and a subsequent recovery, which results in gain variations of ${\delta g}_{d}$ and ${\delta g}_{r}$, respectively, i.e.,

$$\begin{aligned} {\delta g}_{d}=g\left( 1-e^{-\frac{\left\| u \right\|}{E_{G}}} \right)\sim g\frac{\left\| u \right\|}{E_{G}}, \#\left( S2a \right) \end{aligned}$$

$$\begin{aligned} {\delta g}_{r}=\left( g-\Lambda_{0} \right)\delta\left( e^{-\frac{t}{\tau_{e}}} \right)\sim\frac{\left( \Lambda_{0}-g \right)T_{R}}{\tau_{e}}, \#\left( S2b \right) \end{aligned}$$

yielding a ratio of

$$\begin{aligned} \frac{{\delta g}_{r}}{{\delta g}_{d}}\sim\frac{\left( \Lambda_{0}-g \right)}{g}\frac{T_{R}E_{G}}{\tau_{e}\left\| u \right\|}. \#\left( S3 \right) \end{aligned}$$


**Figure S1 | Gain depletion and recovery (GDR) effect in dual-time scales. a.** Equivalent characterization of the gain variation in the fast time scale. ${\delta g}_{d}$ and ${\delta g}_{r}$ are the amounts of gain depletion and recovery in the fast time scale, respectively. $\Delta g$ denotes the effective gain depletion. **b**. Gain variation in dual-time scales. In the fast time scale, the gain $g_{1}\left( T \right)$ that is tailored by the solitons within a soliton crystal begins with a gain coefficient of $g_{0}\left( \tau_{0} \right)$ at the leading edge of the soliton crystal with a duration of [$T_{0}$, $T_{1}$]. The dynamic gain undergoes a recovery process and reaches $g_{0}\left( \tau_{1} \right)$ before the arrival of the next soliton crystal. The saturable loss over the soliton crystal is expressed as $q(E_{p})/T_{R}$, where $q\left( E_{p} \right)$ is the loss from the saturable absorber and $T_{R}$ is the roundtrip time of the short cavity. Ave. and Sat. are short for average and saturable, respectively.

According to Eq. (S3), there exist two regimes:

- For MHz fundamental repetition rates, the time-varying gain undergoes a self-consistent evolution, and it gives rise to ${{\delta g}_{r}}/{{\delta g}_{d}}=1$. In this case, gain depletion can be completely recovered before the arrival of the next soliton, such that the solitons are independent as no gain correlation exists between the solitons.
- For GHz fundamental repetition rates, the gain becomes dynamic for the consecutive solitons, and the gain cannot completely recover before the arrival of the next soliton in a short time interval of sub-ns, i.e., the roundtrip time $T_{R}$ of the GHz fiber laser cavity, leading to ${{\delta g}_{r}}/{{\delta g}_{d}}<1$ and a nonzero $\Delta g$. In this case, the consecutive solitons are dependent, as the behavior of a single soliton is influenced by the other solitons through the dynamic gain.

It should be pointed out that, the dynamic-gain-dominated collective behaviors of the solitons with GHz fundamental repetition rates, particularly in the fashion of soliton crystals, are analogous to the spontaneous assembling of multiple solitons in the harmonically mode-locked fiber laser^23^. Here the dynamic gain manifesting multiscale features can result in versatile phenomena of soliton assembling, broadening the scope of the existing mode-locking model^24–27^. To understand the underlying mechanism of soliton assembling, we introduce the concept of soliton crystal that can well describe the dynamic behaviors of the dense solitons separated by a roundtrip time $T_{R}$of a GHz fiber laser cavity (see **Fig. 1** of the main text). We also extend the mode-locking theory to account for two different time scales of the RE-doped gain fiber, specifically by 1) proposing a new mode-locking theory of GHz fiber lasers involving GDR-driven soliton assembling; 2) bridging the new mode-locking theory with the existing theory through proposing a concept of quasi-single soliton (QSS); 3) understanding the mode-locking dynamics of GHz fiber lasers.

1. Theory of CWML in GHz fiber lasers

A) Gain dynamics in dual-time scales

The uniform formula describing the gain dynamics in RE-doped fiber lasers can be expressed as

$$\begin{aligned} \frac{dN_{2}}{dt}=-\frac{N_{2}}{T_{G}}+\frac{\Gamma}{h\upsilon_{p}A}\sigma_{a\left( p \right)}N_{1}P_{p}+\frac{\Gamma}{h\upsilon_{s}A}\left[ \sigma_{a\left( s \right)}N_{1}-\sigma_{e\left( s \right)}N_{2} \right]P, \#\left( S4a \right) \end{aligned}$$

$$\begin{aligned} g=\Gamma\left[ \sigma_{e\left( s \right)}N_{2}-\sigma_{a\left( s \right)}N_{1} \right], \#\left( S4b \right) \end{aligned}$$

where $N_{1}$ and $N_{2}$ represent the population of the ground state as well as the upper lasing level of ions, with total population of $N=N_{1}+N_{2}$. $T_{G}$ is the lifetime of the upper lasing level. $\Gamma$ is the overlapping factor. $\upsilon_{p}$ and $\upsilon_{s}$ are the (central) frequencies of the pump and signal, respectively. $A$ is the effective mode area of the gain fiber. $\sigma_{a}$ and $\sigma_{e}$ are the absorption and emission cross-sections, respectively. The subscript $p$ and $s$ indicate the cross-sections at the pump and signal wavelengths, respectively. $P_{p}$ and $P$ are the power of the pump and signal, respectively.

By substituting Eq. (S4b) into Eq. (S4a), Eq. (S1) can be written as

$$\begin{aligned} \frac{dg}{dt}=-\frac{g-\Lambda_{0}}{\tau_{e}}-\frac{P}{E_{G}}g, \#\left( S5 \right) \end{aligned}$$

$$with, \tau_{e}=\frac{T_{G}}{1+\frac{\Gamma\sigma_{a\left( p \right)}P_{p}T_{G}}{h\upsilon_{p}A}},$$

$$E_{G}=\frac{h\upsilon_{s}A}{\Gamma\left[ \sigma_{a\left( s \right)}+\sigma_{e\left( s \right)} \right]}, \Lambda_{0}=\tau_{e}\left( \frac{\Gamma^{2}\sigma_{a\left( p \right)}\sigma_{e\left( s \right)}P_{p}N}{h\upsilon_{p}A}-\frac{\Gamma N}{T_{G}} \right),$$

It is worth noting that the pump condition must be considered when evaluating the effective lifetime of the upper level. Eq. (S5) is a general form for studying the gain of optical field. To investigate the rectangular-shape Q-switched mode-locking (RSQSML) involved internal dynamics of soliton assembling, instead, multiscale analysis^28^ must be applied to account for gain depletion and gain recovery processes. To this end, variables in two different time scales are introduced, i.e., a fast time scale $T=t/\eta$ ($\eta\ll1$) that accounts for the soliton-to-soliton variations, and a slow time scale $\tau$ that accounts for the crystal-to-crystal variations, coinciding with the physical time $t$. Given that $g=g_{0}+\eta g_{1}$, we have

$$\begin{aligned} \partial_{t}g=\partial_{\tau}g_{0}+\partial_{T}g_{1}, in \mathcal{O}\left( 1 \right) \#\left( S6 \right) \end{aligned}$$

where $g_{0}$ is irrelevant with the fast time scale $T$, i.e., $\partial_{T}g_{0}=0$. To intuitively understand the parameters in two different time scales, these parameters are indicated in **Fig. S1**, wherein the dynamic gain (i.e., the fast gain depletion and subsequent gain recovery) over a soliton crystal is illustrated. By substituting Eq. (S6) into Eq. (S5), we can rewrite the rate equation by collecting the terms at $\mathcal{O}\left( 1 \right)$, i.e.,

$$\begin{aligned} \partial_{\tau}g_{0}+\partial_{T}g_{1}=-\frac{g_{0}-\Lambda_{0}}{\tau_{e}}-\frac{P\left( \tau,T \right)}{E_{G}}g_{0}, \#\left( S7 \right) \end{aligned}$$

As $g_{1}$ varies much faster than the time interval of soliton crystals, e.g., $\tau_{c}$, we have

$$\begin{aligned} \int_{T_{0}}^{T_{0}+{\tau_{c}}/\eta} \partial_{T}g_{1}dT=\left( -\frac{g_{0}-\Lambda_{0}}{\tau_{e}}-\partial_{\tau}g_{0} \right)\tau_{c}-\frac{g_{0}}{E_{G}}\int_{T_{0}}^{T_{1}} P\left( \tau,T \right)dT=0. \#\left( S8 \right) \end{aligned}$$

Eq. (S8) gives rise to the rate equation of $g_{0}$, i.e.,

$$\begin{aligned} \frac{dg_{0}\left( \tau\right)}{d\tau}=-\frac{g_{0}\left( \tau\right)-\Lambda_{0}}{\tau_{e}}-\frac{g_{0}\left( \tau\right)E_{c}\left( \tau\right)}{E_{G}\tau_{c}}, E_{c}\left( \tau\right)=\int_{T_{0}}^{T_{1}} P\left( \tau,T \right)dT \#\left( S9 \right) \end{aligned}$$

where $E_{c}\left( \tau\right)$ is the energy of a soliton crystal. By further substituting Eq. (S9) into Eq. (S7), the rate equation of $g_{1}$ becomes

$$\begin{aligned} \frac{dg_{1}}{dT}=\frac{g_{0}\left( \tau\right)E_{c}\left( \tau\right)}{E_{G}\tau_{c}}-\frac{g_{0}\left( \tau\right)P\left( \tau,T \right)}{E_{G}}. \#\left( S10 \right) \end{aligned}$$

The gain depletion and recovery depicted by Eq. (S10) can be analyzed by the following scheme:

**Gain depletion:**

$$\begin{aligned} \frac{dg_{1}\left( T \right)}{dT}=-\frac{g_{0}\left( \tau\right)P\left( \tau,T \right)}{E_{G}}, inside the soliton crystal T\in\left[ T_{0}, T_{1} \right]. \#\left( S11a \right) \end{aligned}$$

**Gain recovery:**

$$\begin{aligned} \frac{dg_{1}\left( T \right)}{dT}=\frac{g_{0}\left( \tau\right)E_{c}\left( \tau\right)}{E_{G}\tau_{c}}, outside the soliton crystal. \#\left( S11b \right) \end{aligned}$$

An illustrative description of this equivalent treatment is shown in **Fig. S1a**. In Eq. (S11a), the gain coefficient $g\left( \tau,t \right)$ over the soliton crystal is provided as

$$\begin{aligned} g\left( \tau,t \right)=g_{0}\left( \tau\right)-\frac{g_{0}\left( \tau\right)}{E_{G}}\eta\int_{T_{0}}^{T} P\left( \tau,T^{'} \right)dT^{'}=g_{0}\left( \tau\right)\left( 1-\frac{1}{E_{G}}\int_{\tau_{0}}^{t} P\left( \tau,t^{'} \right)dt^{'} \right), \#\left( S12 \right) \end{aligned}$$

and its unsaturated form is

$$\begin{aligned} g\left( \tau,t \right)=g_{0}\left( \tau\right)exp\left( -\frac{1}{E_{G}}\int_{\tau_{0}}^{t} P\left( \tau,t^{'} \right)dt^{'} \right). \#\left( S13 \right) \end{aligned}$$

For non-negligible ${\delta g}_{r}$, the effective gain depletion is defined as $\Delta g$, and a higher pump power corresponds to a smaller $g_{0,}$ as shown in **Fig. 1** of the main text. $g_{0}$ decreases as the pump power increases. With a soliton crystal energy $E_{c}\left( \tau\right)$ and normalized envelope function $f\left( t \right)$, the laser power $P\left( \tau,t \right)$ in Eq. (S13) can be expressed as

$$\begin{aligned} P\left( \tau,t \right)=\sum_{i} E_{c}\left( \tau\right)f\left( t-i\tau_{c} \right), \int_{0}^{\tau_{c}} f\left( t-i\tau_{c} \right)dt=1. \#\left( S14 \right) \end{aligned}$$

Then, the dynamic gain over the soliton crystal can be equivalently interpreted as an averaged gain $g_{c}\left( \tau\right)$, i.e.,

$$g_{c}\left( \tau\right)=\int_{\tau_{0}}^{{\tau_{0}+\tau}_{c}} g\left( \tau,t \right)f\left( t \right)dt=g_{0}\left( \tau\right)\int_{0}^{\tau_{c}} f\left( t \right)exp\left( -\frac{E_{c}\left( \tau\right)}{E_{G}}\int_{0}^{t} f\left( t^{'} \right)dt^{'} \right)dt$$

$$\begin{aligned} =g_{0}\left( \tau\right)\frac{1-exp\left( -{E_{c}\left( \tau\right)}/{E_{G}} \right)}{{E_{c}\left( \tau\right)}/{E_{G}}}. \#\left( S15 \right) \end{aligned}$$

B) Soliton crystals formed from soliton assembling

The lumped linear loss $q_{l}$ and saturable absorption $q$, featured with instantaneous response, are averaged over the roundtrip time $T_{R}$, while the averaged gain $g_{c}\left( \tau\right)$ is uniformly distributed across the soliton crystal. The rate equation describing the energy of the soliton crystal is written as

$$\begin{aligned} \frac{dE_{c}\left( \tau\right)}{d\tau}=\left[ g_{c}\left( \tau\right)-\frac{q_{l}}{T_{R}}-\frac{q}{T_{R}} \right]E_{c}\left( \tau\right) \#\left( S16 \right) \end{aligned}$$

$$\mathrm{with}q=\frac{q_{0}\left[ 1-exp\left( -{E_{c}\left( \tau\right)T_{R}}/{E_{a}\Delta T} \right) \right]}{{E_{c}\left( \tau\right)T_{R}}/{E_{a}\Delta T}},$$

where the pulse energy $E_{p}$ is substituted by ${E_{c}\left( \tau\right)T_{R}}/{\Delta T}$ as the soliton crystal has a flat-top envelope.$\Delta T$ is the duration of the soliton crystal. $q_{0}$ and $E_{a}$ are the modulation depth and saturation energy of SESAM. Eq. (S16) is then rewritten and combined with the rate equation (S9) of $g_{0}\left( \tau\right)$ to derive the governing equations for soliton assembling, yielding

$$\begin{aligned} T_{R}\frac{dE_{c}}{d\tau}=\left[ 2g_{0}L\frac{1-exp\left( -{E_{c}}/{E_{G}} \right)}{{E_{c}}/{E_{G}}}-q_{l}-\frac{q_{0}\left[ 1-exp\left( -{E_{c}T_{R}}/{E_{a}\Delta T} \right) \right]}{{E_{c}T_{R}}/{E_{a}\Delta T}} \right]E_{c}, \end{aligned}$$

$$\begin{aligned} \frac{dg_{0}}{d\tau}=-\frac{g_{0}-\Lambda_{0}}{\tau_{e}}-\frac{g_{0}E_{c}}{E_{G}\tau_{c}}. \#\left( S17 \right) \end{aligned}$$

According to Eq. (S17), we obtain the corresponding ordinary differential equations (ODEs) for the perturbations of $E_{c}$ and $g_{0}$ nearby the fixed point ($\bar{E_{c}}$, $\bar{g_{0}}$), i.e.,

$$T_{R}\frac{d{\delta E}_{c}}{d\tau}=\left( -q_{l}-q_{0}e^{-{\bar{E_{c}}T_{R}}/{E_{a}\Delta T}}+2\bar{g_{0}}Le^{-{\bar{E_{c}}}/{E_{G}}} \right){\delta E}_{c}+2E_{G}L\left( 1-e^{-{\bar{E_{c}}}/{E_{G}}} \right)\delta g_{0},$$

$$\begin{aligned} T_{R}\frac{d\delta g_{0}}{d\tau}=-\frac{T_{R}\bar{g_{0}}}{E_{G}\tau_{c}}{\delta E}_{c}-\left( \frac{T_{R}}{\tau_{e}}+\frac{T_{R}\bar{E_{c}}}{E_{G}\tau_{c}} \right)\delta g_{0}. \#\left( S18 \right) \end{aligned}$$

The corresponding Jacobi matrix is calculated as

$$M_{J}=\left( \begin{matrix} -q_{l}-q_{0}e^{-{\bar{E_{c}}T_{R}}/{E_{a}\Delta T}}+2\bar{g_{0}}Le^{-{\bar{E_{c}}}/{E_{G}}} & 2E_{G}L\left( 1-e^{-{\bar{E_{c}}}/{E_{G}}} \right) \\ -\frac{T_{R}\bar{g_{0}}}{E_{G}\tau_{c}} & -\left( \frac{T_{R}}{\tau_{e}}+\frac{T_{R}\bar{E_{c}}}{E_{G}\tau_{c}} \right) \end{matrix} \right).$$

Consequently, the soliton crystals survive to generate stable continuous soliton train when

$$\begin{aligned} tr\left( M_{J} \right)<0. \#\left( S19 \right) \end{aligned}$$

In conjunction with the relation of $dE_{c}/d\tau=0$, Eq. (S19) can be rewritten as

$$-\left( \frac{T_{R}}{\tau_{e}}+\frac{T_{R}\bar{E_{c}}}{E_{G}\tau_{c}} \right)+\left( q_{l}+q_{0}\frac{1-e^{-{\bar{E_{c}}T_{R}}/{E_{a}\Delta T}}}{{\bar{E_{c}}T_{R}}/{E_{a}\Delta T}} \right)\frac{\bar{E_{c}}e^{-{\bar{E_{c}}}/{E_{G}}}}{E_{G}\left( 1-e^{-{\bar{E_{c}}}/{E_{G}}} \right)}$$

$-q_{l}-q_{0}e^{-{\bar{E_{c}}T_{R}}/{E_{a}\Delta T}}<0$. $(S20)$

C) Criterion of CWML for transiting from soliton crystals to a stable continuous soliton train

Based on Eq. (S20), we can derive a criterion of CWML, by considering two aspects:

- The first term, i.e., $\left( {T_{R}}/{\tau_{e}}+{T_{R}\bar{E_{c}}}/{E_{G}\tau_{c}} \right)$, is small enough when $T_{R}\ll\tau_{e}$ and $T_{R}\ll\tau_{c}$ (referring to the typical values of the key parameters provided in **Table S2**).
- We substitute the functions of $q_{0}$ and $\bar{g_{0}}$ for the linear loss $q_{l}$, namely,

$$q_{l}=2\bar{g_{0}}L\frac{1-exp\left( -{\bar{E_{c}}}/{E_{G}} \right)}{{\bar{E_{c}}}/{E_{G}}}-\frac{q_{0}\left[ 1-exp\left( -{\bar{E_{c}}T_{R}}/{E_{a}\Delta T} \right) \right]}{{\bar{E_{c}}T_{R}}/{E_{a}\Delta T}}.$$

Then, Eq. (S20) enables a direct comparison between saturable loss and gain, i.e.,

$$2\bar{g_{0}}L\left( e^{-{\bar{E_{c}}}/{E_{G}}}-\frac{1-exp\left( -{\bar{E_{c}}}/{E_{G}} \right)}{{\bar{E_{c}}}/{E_{G}}} \right)$$

$$<q_{0}\left( e^{-{\bar{E_{c}}T_{R}}/{E_{a}\Delta T}}-\frac{\left[ 1-exp\left( -{\bar{E_{c}}T_{R}}/{E_{a}\Delta T} \right) \right]}{{\bar{E_{c}}T_{R}}/{E_{a}\Delta T}} \right).$$

$$(S21)$$

Considering $\bar{E_{c}}\ll E_{G}$, the left-hand side of Eq. (S21) can be reduced to

$$\begin{aligned} \lim_{{\bar{E_{c}}}/{E_{G}}\to0} 2\bar{g_{0}}L\left( e^{-{\bar{E_{c}}}/{E_{G}}}-\frac{1-exp\left( -{\bar{E_{c}}}/{E_{G}} \right)}{{\bar{E_{c}}}/{E_{G}}} \right)=-\frac{\bar{g_{0}}L\bar{E_{c}}}{E_{G}}. \#\left( S22 \right) \end{aligned}$$

Assuming a balance between $\bar{g_{0}}L$ (i.e., half gain in the leading part of the soliton crystal) and $q_{0}$ (nearly half cavity loss with saturated SESAM), i.e., $\bar{g_{0}}L= q_{0}$, we have

$$\begin{aligned} \frac{\bar{E_{c}}}{E_{G}}>\frac{\left[ 1-exp\left( -{\bar{E_{c}}T_{R}}/{E_{a}\Delta T} \right) \right]}{{\bar{E_{c}}T_{R}}/{E_{a}\Delta T}}-e^{-{\bar{E_{c}}T_{R}}/{E_{a}\Delta T}}. \#\left( S23 \right) \end{aligned}$$

As it approaches the CWML state, i.e., $\Delta T\to\tau_{c}$, Eq. (S23) becomes

$$\begin{aligned} \lim_{\Delta T\to\tau_{c}}\frac{\bar{E_{c}}}{E_{G}}=\underset{Y_{1}}{\underbrace{\frac{E_{p}f_{R}}{E_{G}f_{c}}}}>\underset{Y_{2}}{\underbrace{\frac{\left[ 1-exp\left( -{E_{p}}/{E_{a}} \right) \right]}{{E_{p}}/{E_{a}}}-e^{-{E_{p}}/{E_{a}}}}}. \#\left( S24 \right) \end{aligned}$$

For strongly saturated SESAMs, i.e., ${E_{p}}/{E_{a}}\gg1$, it results in

$$\begin{aligned} E_{p}^{2}>\frac{f_{c}}{f_{R}}E_{a}E_{G}, \#\left( S25 \right) \end{aligned}$$

which is analogous to the existing mode-locking theory^29^. Without the assumption of $\bar{g_{0}}L= q_{0}$, a more general form of the criterion is given as

$$\begin{aligned} E_{p}^{2}>\frac{q_{0}f_{c}}{\bar{g_{0}}Lf_{R}}E_{a}E_{G}, \#\left( S26 \right) \end{aligned}$$

where, $f_{c}$ is the repetition rate of soliton crystals. By referring to typical values summarized in **Table S2**, the CWML threshold of pulse energy $E_{p}$ is estimated to be several tens of pJ, e.g., ~26.7 pJ, which is in good agreement with the critical energy of ~24 pJ achieved in the experiment. The key parameters used in the calculation include repetition rates of 0.5 MHz and 21 GHz for the soliton crystal and individual soliton, respectively, and gain saturation energy of 4 μJ.

**Table S2 | Key parameters used in the calculation**

| **Parameter** | **Notation** | **Typical value (range)** |
| --- | --- | --- |
| Modulation depth | $q_{0}$ | 0.03 |
| Saturation energy of SESAM | $E_{a}$ | 7.5 pJ |
| Gain saturation energy | $E_{G}$ | 1~5 μJ |
| Effective upper-level lifetime | $\tau_{e}$ | Pump-dependent, 10~50 μs |
| Effective mode area | $A$ | 1.12×10^-11^ m^2^ |
| Overlapping factor | $\Gamma$ | 0.8 |
| Absorption cross-section at pump wavelength | $\sigma_{a\left( p \right)}$ | 2×10^-24^ m^2^ |
| Cross-section in the signal band | $\sigma_{a\left( s \right)}+\sigma_{e\left( s \right)}$ | 4.9×10^-25^ m^2^ |
| Pulse energy* | $E_{p}$ | 10~50 pJ* |
| Repetition rate of soliton crystal* | $f_{c}$ | 50 kHz ~1 MHz* |
| Energy of the soliton crystal | $E_{c}$ | Variable, ~1 μJ |

*Consistent with the experiment.

The above analysis implies that CWML with a pulse energy closer to $E_{p0}$ tends to have a higher pump threshold, as illustrated by **Figs. S2a,b**. By expanding the first derivative of $Y_{2}$as second-order Taylor polynomial, i.e.,

$$\left. \frac{d\left( \left[ 1-exp\left( -x \right) \right]/x-e^{-x} \right)}{dx} \right|_{x=x_{0}}=0$$

$\Rightarrow x_{0}^{2}+4x_{0}-12=0, \mathrm{with} x_{0}={E_{p0}}/{E_{a}}.$ (S27)

There is a simple approximation $E_{p0}\sim2E_{a}=15 \mathrm{pJ}$. We summarize the experimental results of mode-locking with fundamental repetition rates of 4.9^15^, 12.5^16^, and 16.7 GHz (the table of **Fig. S2**), and find that the pump threshold of CWML increases as critical pulse energy approaches the analytical prediction of $E_{p0}$, i.e., 15 pJ (**Figs. S2a,b**).

Figure S2 | Functions of $\boldsymbol{Y}_{\boldsymbol{2}}$ and ${\boldsymbol{E}_{\boldsymbol{c}}}/{\boldsymbol{E}_{\boldsymbol{G}}}$. a,b. Functions of$\boldsymbol{Y}_{\boldsymbol{2}}$ and ${\boldsymbol{E}_{\boldsymbol{c}}}/{\boldsymbol{E}_{\boldsymbol{G}}}$ with varying pulse energy and pump power, respectively. Experimental results with different fundamental repetition rates (4.9, 12.5, and 16.7 GHz) are also summarized in the table, and indicated in a.

- 1. **Bridging the new theory with existing mode-locking theory through proposing a concept of quasi-single soliton (QSS)**

To bridge the criterion of CWML in passively mode-locked GHz fiber lasers with the existing mode-locking theory, we express Eq. (S26) in the forms of

$$\begin{aligned} C\mathrm{lassical} criterion: E_{p\left( single \right)}^{2}>q_{0}E_{a}E_{G}, \#\left( S28a \right) \end{aligned}$$

$$\begin{aligned} E_{p}^{2}>q_{0}E_{a}E_{G,eff} , \text{ }\mathrm{with}E_{G,eff}=E_{G}\frac{f_{c}}{\bar{g_{0}}Lf_{R}}, \text{ }\#\left( S28b \right) \end{aligned}$$

$$\begin{aligned} \left( N_{sc}E_{p} \right)^{2}>q_{0}E_{a}E_{G},\text{ }\mathrm{with}T_{SC}=T_{R}\sqrt{\frac{\bar{g_{0}}Lf_{R}}{f_{c}}}. \#\left( S28c \right) \end{aligned}$$

We define an effective gain saturation energy $E_{G,eff}$ and strongly-correlated (SC) length $T_{SC}$, based on which we introduce the concept of QSS to characterize the collective behaviors of soliton assembling driven by the dynamic gain, and we can understand the GDR-mediated mode-locking as below:

- The soliton crystals generated by soliton assembling can be regarded as a sequence of QSS with equivalent energy of $E_{p}$, and its SC length $T_{SC}$ corresponds to multiple roundtrip times of the GHz fiber laser cavity. Then, as an elementary unit, the QSS experiences an effective gain saturation determined by $E_{G,eff}$.

The concept of QSS enables the bridge between the new mode-locking theory of GHz fiber lasers with the existing mode-locking theory, giving rise to a modified form of classical criterion, i.e.,

$$\begin{aligned} \frac{T_{SC}}{\tau_{e}}+\frac{E_{p}}{E_{G,eff}}>q_{0}\left( \frac{\left[ 1-exp\left( -{E_{p}}/{E_{a}} \right) \right]}{{E_{p}}/{E_{a}}}-e^{-{E_{p}}/{E_{a}}} \right).\#\left( S29 \right) \end{aligned}$$

In the meantime, such a bridge facilitates us to extend the numerical model of the QSS for studying the dynamics of soliton crystals through

$$\begin{aligned} \frac{\partial u_{i}}{\partial z}=\left( -i\frac{\beta_{2}}{2}+\frac{g\left( z,\tau_{i} \right)}{\Omega_{g}^{2}} \right)\frac{\partial^{2}u_{i}}{\partial T^{2}}+i\gamma\left| u_{i} \right|^{2}u_{i}+g\left( z,\tau_{i} \right)u_{i}, \#\left( S30a \right) \end{aligned}$$

$$\begin{aligned} \frac{dq}{dT}=-\frac{q-q_{0}}{\tau_{a}}-\frac{\left| u \right|^{2}}{E_{a}}q, \#\left( S30b \right) \end{aligned}$$

$$\begin{aligned} \frac{\partial g\left( z,\tau\right)}{\partial\tau}=-\frac{g\left( z,\tau\right)-\Lambda_{0}}{\tau_{e}}-\frac{\left\| u \right\|^{2}}{E_{G,eff}T_{SC}}g\left( z,\tau\right), \text{ }\#\left( S30c \right) \end{aligned}$$

where $u_{i}$ is the optical field of the QSS. $\beta_{2}$, $\Omega_{g}$, and $\gamma$ are the second-order dispersion, gain bandwidth, and nonlinearity of gain fiber, respectively. $\tau_{a}$ is the relaxation time of the saturable absorber. For numerical calculation, $u_{i}$ is defined in a time window of [-${T_{R}}/2$, ${T_{R}}/2$] (identical with the roundtrip time of the GHz fiber laser cavity) to render an equivalent energy of $E_{p}$, and the propagation distance along $z$ corresponds to the roundtrip time $T_{R}$. For solving Eq. (S30c), the time step is set to $\tau_{i}=iT_{SC}$ (multiple roundtrip times of the GHz fiber laser cavity).

Figure S3 | Typical dynamics of RSQSML in the simulation. a. Intensity evolution (left) and trajectory in the phase plane (right). The corresponding SC length used in the simulation is 3.3 ns, and delay roundtrip number is $N_{D}=5$. b. Corresponding temporal (left) and spectral (right) evolutions.

By changing the SC length $T_{SC}$, distinctive evolutionary behaviors of QSSs are obtained in the numerical simulation, including rectangular-shape and Gaussian-shape Q-switched mode-locking (i.e., RSQSML and GSQSML), as shown in **Figs. 3** and **4** of the main text, consistent with the experiment (i.e., the case with a fundamental repetition rate of 11 GHz). With relatively long $T_{SC}$, there is sufficient time for the depleted gain to recover, which facilitates a steady state, as indicated in **Fig. S3a**. Other characteristics, e.g., temporal and spectral evolutions, are shown in **Fig. S3b**.

When the fundamental repetition rate increases to multi-GHz, the gain can no longer be retained due to the reduction of the SC length. In this case, an oscillatory evolution landscape is presented, particularly in the form of GSQSML (left panel of **Fig. S4a**). In the oscillatory evolution, we find that the variation of the peak power is positively correlated with that of energy (right panel of **Fig. S4a**). The spatiotemporal and spatiospectral evolution landscapes are shown in **Fig. S4b**. It exhibits similar features as that of breathing solitons observed in other nonlinear platforms^30,31^. However, it is worth noting that there is a substantial difference: the oscillatory evolution here is initiated by the gain dynamics, while such time-varying gain is not incorporated for generating breathing solitons in other nonlinear platforms^32^.

Figure S4 | Typical dynamics of GSQSML in the simulation. a. Intensity evolution (left) and trajectory in the phase plane (right). Corresponding SC length used in the calculation is 1.4 ns, and delay roundtrip number $N_{D}=5$. b. Corresponding temporal (left) and spectral (right) evolutions.

- 1. **Understanding the mode-locking dynamics of GHz fiber lasers**

In this section, we understand the mode-locking dynamics of GHz fiber lasers based on the above framework, as depicted in **Fig. S5**. We first derive the conditions for RSQSML and GSQSML via the concept of QSS by leveraging Eq. (S29). For convenience, these two inequalities are termed as conditions for RSQSML and GSQSML before successful CWML, respectively, i.e.,

$$\begin{aligned} \mathbf{RSQSML}:\frac{T_{SC}}{\tau_{e}}+\frac{E_{p}}{E_{G,eff}}>q_{0}\left( \frac{\left[ 1-exp\left( -{E_{p}}/{E_{a}} \right) \right]}{{E_{p}}/{E_{a}}}-e^{-{E_{p}}/{E_{a}}} \right),\text{ }\#(S31a) \end{aligned}$$

$$\begin{aligned} \mathbf{GSQSML}:\frac{T_{SC}}{\tau_{e}}+\frac{E_{p}}{E_{G,eff}}<q_{0}\left( \frac{\left[ 1-exp\left( -{E_{p}}/{E_{a}} \right) \right]}{{E_{p}}/{E_{a}}}-e^{-{E_{p}}/{E_{a}}} \right).\#(S31b) \end{aligned}$$

The key parameters used in the study are also indicated in **Table S3**. For better understanding, we discuss the two distinguishing dynamics of Q-switched mode-locking (QSML).

**Rectangular-shape Q-switched mode-locking (RSQSML):** The QSS likely reaches equilibrium energy at a lower level of pump power. Since $g_{0}$ is larger for smaller soliton crystal energy and larger $\Delta g$ [see the description of Eq. (S13)] at a lower level of pump power, it results in a longer $T_{SC}$ [see Eq. (S28c)] that ensures the satisfaction of the requirement described by Eq. (S31a). Thus, the QSSs are stable to generate soliton crystals with flat-top envelopes (see **Fig. 3a** in the main text), yielding RSQSML dynamics.

**Gaussian-shape Q-switched mode-locking (GSQSML):** For a shorter $T_{SC}$, a simultaneous increase of the effective gain saturation energy $E_{G,eff}$ renders the requirement described by Eq. (S31b). It then prevents stable QSSs, as the solitons are unstable according to the classical criterion of CWML. Thus, the resultant dynamics exhibit similar features with the classical QSML that has a Gaussian-shape envelope, as manifested in **Fig. 4b** in the main text.

Figure S5 | Flow chart for understanding the mode-locking dynamics of GHz fiber lasers.

Table S3 | Key parameters used in the case with a fundamental repetition rate of 21 GHz

| **Parameter** | **Value** |
| --- | --- |
| Pulse repetition rate ($f_{R}$) | 21 GHz |
| Modulation depth of SESAM ($q_{0}$) | 0.03 |
| Repetition rate of the soliton crystals ($f_{c}$) | 300~400 kHz |
| Gain saturation energy ($E_{G}$) | 4.26 μJ |
| Effective saturation energy ($E_{G,eff}$) | 1.4 nJ (for RSQSML)  4.7 nJ (for GSQSML) |
| Effective upper-level lifetime ($\tau_{e}$) | 14 μs |
| Number of solitons in SC length ($N_{SC}$) | 55 (for RSQSML)  30 (for GSQSML) |
| SC length ($T_{SC}$) | 2.6 ns (for RSQSML)  1.4 ns (for GSQSML) |

Supplementary Note 3: Time-lens magnification measurement system

The experimental setup of the time-lens magnification measurement system is shown in **Fig. S6a**. It is implemented through the four-wave mixing (FWM) process pumped by linearly chirped pulses. In the pump path, a mode-locked fiber laser (MLFL) with a tunable repetition rate of around 19.6 MHz is employed as the pump source. The pump pulses are first chirped by a spool of dispersion compensating fiber (DCF1) and then filtered by a bandpass filter at 1550 nm (BPF1, 13 nm bandwidth). The chirped pump pulses are amplified by a C-band erbium-doped fiber amplifier (EDFA1) to about 40 mW. In the signal path, the signal under test (SUT), i.e., a mode-locked fiber laser with a central wavelength of ~1565 nm and fundamental repetition rate of 11 GHz, is used, and first goes through an optical coupler (OC1). The 10% port of OC1 is used for monitoring through a high-speed photodetector (PD1, Newport 818-BB-51F, 12.5 GHz bandwidth) and a standard real-time oscilloscope (Osci, Keysight DSOV204A, 20 GHz bandwidth). The signal extracted from the 90% port is then amplified by EDFA2 to about 45 mW and subsequently chirped by DCF2. The chirped SUT is then combined with the chirped pump pulses through 10% and 90% input ports of OC2, respectively. The 10% output port of OC2 is used for the synchronization between the SUT and the pump through PD2 (Newport 818-BB-51F, 12.5 GHz bandwidth) and a radio-frequency spectrum analyzer (RSA, Rohde & Schwarz FSWP26, 26.5 GHz bandwidth). The combined beam is sent to a spool of 10-m highly nonlinear fiber (HNLF, YOFC NL-1550-Zero). An idler at around 1535 nm is generated through the FWM process inside the HNLF, as shown in **Fig. S6b**, which is measured by an optical spectrum analyzer (OSA, YOKOGAWA AQ6370D). To maximize the FWM efficiency, the states of polarization of both SUT and chirped pump beams are individually controlled by two polarization controllers (PCs). The idler ranging from 1532 nm to 1538 nm is filtered by another bandpass filter (BPF2, ~6 nm bandwidth) with an average power of around 20 μW and then passes through DCF3, which is recorded by PD3 (Newport 1544-B, 12.5 GHz bandwidth) and real-time oscilloscope.

Overall, the system functions as a temporal analogue of a spatial single-lens imaging system. Three spools of DCFs (DCF1, DCF2 and DCF3) provide pump group delay dispersion (GDD) of -39 ps·nm^-1^ ($D_{p}$), input GDD of -19 ps·nm^-1^ ($D_{in}$) and output GDD of -760 ps·nm^-1^ ($D_{out}$), respectively. Therefore, a temporal imaging condition is satisfied, i.e.,

$$\begin{aligned} \frac{1}{D_{in}}=\frac{2}{D_{p}}+\frac{1}{D_{out}},\#\left( S32 \right) \end{aligned}$$

and the temporal magnification ratio $D_{out}/D_{in}$ is around 40. To experimentally characterize the magnification ratio of the time-lens system, we measured a dual-pulse waveform with 36-ps temporal separation by a commercial autocorrelator (APE Pulsecheck USB 50, 50 ps scan range) and the time-lens magnification measurement system, respectively, as shown in **Fig. S6c**. Due to the limited record length of the autocorrelator, we only capture a single-sided profile of the whole symmetric autocorrelation trace.

**Figure S6 | Experimental setup and basic performance of the time-lens magnification measurement system. a.** Schematic diagram of the time-lens magnification measurement system. SUT, signal under test. MLFL, mode-locked fiber laser. OC, optical coupler. EDFA, erbium-doped fiber amplifier. DCF, dispersion compensating fiber. PC, polarization controller. HNLF, highly nonlinear fiber. PD, photodetector. CH, channel. Osci, oscilloscope. RSA, radio-frequency spectrum analyzer. **b.** Optical spectrum of the optical signal after the four-wave mixing process in the HNLF. **c.** Autocorrelation trace (top, AC) and time-lens magnification measurement (bottom, TL). **d.** Waveforms measured by a standard real-time oscilloscope (blue) and time-lens magnification system (red) in the buildup process. The time delay between these two measurements is 34.55 $\mu$s, as indicated.

For a GDD of -39 ps·nm^-1^ and a bandwidth of ~13 nm in the pump, the recording time window of the time-lens magnification measurement system can be calculated as 39 ps·nm^-1^$\times$13 nm $=$ 507 ps. Please note that, the actual time window during the measurement is shorter than the calculated value due to the presence of the limited bandwidth of BPF2 (i.e., 6 nm). In this situation, only one pulse (instead of two or three pulse individuals) can be captured, as showcased by the results in the main text (i.e., **Figs. 3c** and **4d**). The temporal resolution $\tau_{res}$ of the time-lens system can be estimated as $\tau_{res}=\tau_{p}/\sqrt{2}\approx272$ fs, where $\tau_{p}$ is the transform-limited duration of the pump pulse.

In the experiment, to minimize the influence of walking-off between the SUT and the pump in the time-lens magnification measurement, the repetition rate difference between the SUT and pump is fine-tuned for a short-term synchronization. In our case, the repetition rate of the multi-GHz fiber laser $f_{rep,SUT}$ is comparable with the harmonic frequency of the pump pulse, i.e., $Nf_{rep,pump}$ (*N* is the order of the harmonics). To ensure a relatively synchronized sampling, a small frequency difference $\Delta f$ defined by $\Delta f=\left| f_{rep,SUT}-Nf_{rep,pump} \right|$ should be guaranteed, e.g., $\Delta f<10$ kHz here. Finally, to calibrate the time delay between a direct measurement (CH1) and time-lens measurement (CH2), two-channel waveforms are simultaneously captured by the standard real-time oscilloscope in the buildup process of the multi-GHz mode-locked fiber laser. As depicted in **Fig. S6d**, a time delay of 34.55 $\mu$s is measured.

Supplementary Note 4: Tolerable range of the pump threshold of CWML

Based on the proposed mode-locking theory, a phenomenological method can be used to predict the pump threshold of CWML, i.e., $P_{p\_CWML}$. To this end, the criterion Eq. (S24) is rewritten as

$$\begin{aligned} \frac{E_{p}f_{R}}{E_{G}f_{c}}-\frac{\left[ 1-exp\left( -{E_{p}}/{E_{a}} \right) \right]}{{E_{p}}/{E_{a}}}+e^{-{E_{p}}/{E_{a}}}=0. \#\left( S33a \right) \end{aligned}$$

In the state approaching CWML,

$$\begin{aligned} Y_{1}\to\left( {E_{c}}/{E_{G}} \right)_{cri},\#\left( S33b \right) \end{aligned}$$

$$\left( {E_{c}}/{E_{G}} \right)_{cri}=\frac{\eta_{sl}\left( P_{p}-P_{th} \right)}{q_{DF}f_{c}E_{G}}, (S33c)$$

where $\eta_{sl}$, $P_{th}$, and $q_{DF}$ are the slope efficiency, pump threshold of lasing, and output ratio, respectively. The prediction includes three steps:

- First, for soliton crystals with a repetition rate of $f_{c}$, the critical pulse energy $E_{p}\left( f_{c} \right)$ is calculated by Eq. (33a), through which the critical value $Y_{2,\text{ }cri}$ is also obtained;
- Second, the critical pulse energy $E_{p}\left( f_{c} \right)$ determines the value of the corresponding $\left( {E_{c}}/{E_{G}} \right)_{cri}$ in the CWML state via Eq. (S33b);
- Third, Eq. (S33c) is utilized to calculate the pump threshold of CWML $P_{p\_CWML}\left( f_{c} \right)$, i.e.,

$$P_{p\_CWML}\left( f_{c} \right)=\frac{\left( {E_{c}}/{E_{G}} \right)_{cri}q_{DF}f_{c}E_{G}}{\eta_{sl}}+P_{th}. (S34)$$

For intuitive understanding, the steps described above are illustrated in **Fig. S7**. It is worth noting that, the predicational calculation is semi-phenomenological as here the values of slope efficiency $\eta_{sl}$, lasing threshold $P_{th}$, and repetition rate of soliton crystal $f_{c}$ are experimentally determined. Given that the GDR effect in the slow time scale is not sufficient to strongly lock the temporal spacing between the soliton crystals, and the repetition rate of soliton crystals ($f_{c}$) may change even at the same pump power or when pump power increases, we statistically analyze the CWML threshold $P_{p\_CWML}\left( f_{c} \right)$ by accordingly presenting its standard deviation as the tolerable range in the main text (see **Fig. 5a**).

Figure S7 | Steps for calculating the pump threshold of CWML.

Supplementary Note 5: Experimental setups and results of the GHz fiber lasers with different fundamental repetition rates

1. A mode-locked fiber laser with a fundamental repetition rate of 21 GHz

**Figure S8a** illustrates the experimental setup of a fiber laser with a fundamental repetition rate of 21 GHz. The GHz fiber laser has a Fabry-Pérot (FP) cavity that consists of a SESAM (Batop GmbH SAM-1550-7-10 ps), a dielectric film (DF), and a piece of 4.7-mm homemade heavily Er^3+^/Yb^3+^ co-doped phosphate fiber (EYDF) with a net gain coefficient of 9.1 dB·cm^-1^. The SESAM has a modulation depth of 3% and a saturation fluence of 15 μJ·cm^-2^ that facilitates the onset of mode-locking. In addition to the SESAM, the fiber-type DF is used as the other intracavity reflector as well as the output coupler, which has a transmittance of 99.5% at 974 nm (i.e., pump) and a high reflection of ~99% at 1530-1570 nm (i.e., signal). The gain fiber is pumped by a 974-nm single-mode laser diode (SM-LD) via a 974/1550 nm wavelength division multiplexer (WDM). For a long-term stable operation, a temperature controller (TC) and a polarization controller (PC) are used for thermal and polarization control, respectively. An isolator (ISO) is spliced to the WDM to prevent back reflection.

Figure S8 | Schematic diagram of a fiber laser with a fundamental repetition rate of 21 GHz. a. Experimental setup of the 21-GHz fiber laser. SESAM, semiconductor saturable absorber mirror. EYDF, Er^3+^/Yb^3+^ co-doped phosphate fiber. DF, dielectric film. TC, temperature controller. PC, polarization controller. WDM, wavelength division multiplexer. SM-LD, single-mode laser diode. ISO, isolator. b. Configuration of the external amplifier. EDF, erbium-doped fiber.

An erbium-doped fiber amplifier (EDFA) is employed to externally amplify the pulse signal for characterization, as shown in **Fig. S8b**, which includes a 2.5-m long erbium-doped fiber (EDF) forward pumped by another SM-LD via a 974/1550 nm WDM.

Figure S9 | Experimental results of the mode-locked fiber laser with a fundamental repetition rate of 21 GHz. a. Output power as a function of the pump power. Inset shows the oscilloscopic trace of the CWML pulses at time spans of 500 ps (top) and 1 μs (bottom), respectively. b. Stability characterization of the output power, including power evolution over 30 mins (left) and histogram of the power evolution (right) that exhibits a relative standard deviation (RSD) of 1.3%. c. Autocorrelation trace and its sech^2^-fitting.

**Figure S9a** presents the output power of the mode-locked GHz fiber laser as a function of the pump power. RSQSML is realized at a low level of pump power and transited to GSQSML when the pump power is over 80 mW (but less than 85 mW). The CWML is finally achieved at a pump power of more than 85 mW. The inset of **Fig. S9a** shows the oscilloscopic trace of the CWML pulses. It is noted that the oscilloscopic trace is in the shape of sinewave due to the limited bandwidth of the standard real-time oscilloscope used in the experiment. To evaluate the stability of the mode-locked GHz fiber laser, its output power is monitored for 30 minutes, as shown in **Fig. S9b**, and its histogram predicts a relative standard deviation of 1.3%. **Fig. S9c** illustrates the autocorrelation trace of the CWML pulses, which indicates a pulse duration of 2.6 ps, assuming a sech^2^ pulse shape.

1. A mode-locked fiber laser with a fundamental repetition rate of 11 GHz

The mode-locked fiber laser with a fundamental repetition rate of 11 GHz has a similar structure like **Fig. S8a**. It presents similar mode-locking dynamics with that of the 21-GHz fiber laser. As the pump power increases, RSQSML is first realized at a low level of pump power and transited to GSQSML at the pump power of 90 mW. The CWML is realized when the pump power reaches 92.5 mW. **Fig. S10a** presents the pulse train of CWML with a span of 1 μs. It is noted that the oscilloscopic trace is in the shape of sinewave due to the limited bandwidth of the measurement system. The optical spectrum of the CWML pulses is shown in **Fig. S10b**, with a 3-dB bandwidth of 1.5 nm centered at 1565.7 nm. The corresponding longitudinal mode spacing of 0.09 nm is clearly shown (inset of **Fig. S10b**). **Fig. S10c** illustrates the RF spectrum measured with a resolution bandwidth (RBW) of 1 kHz, which indicates a repetition rate of 11 GHz. The top panel of **Fig. S10d** illustrates the single sideband phase noise ranging from 10 Hz to 1 MHz and the integrated timing jitter in the frequency range from 10 Hz to 1 MHz is about 13.4 ps, as shown on the bottom panel of **Fig. S10d**.

Figure S10 | Experimental results of the mode-locked fiber laser with a fundamental repetition rate of 11 GHz. a. Oscilloscopic trace of the CWML pulses at time spans of 1 ns (top) and 1 μs (bottom), respectively. Int., intensity. b. Optical spectrum. The inset depicts the closeup of the optical spectrum that manifests that longitudinal mode spacing of ~0.09 nm. c. RF spectrum measured with a resolution bandwidth (RBW) of 1 kHz. Inset shows the RF spectrum over a wider span ranging from 0 to 26.5 GHz. d. Phase noise (top) and integrated timing jitter (bottom). P. N., phase noise.

1. A frequency-stabilized mode-locked fiber laser with a fundamental repetition rate of 4.6 GHz

**Figure S11** illustrates the configuration of a frequency-stabilized mode-locked fiber laser with a fundamental repetition rate of 4.6 GHz. Compared with the cavity that has a fundamental repetition rate of 21 GHz, a longer EYDF (21.4 mm in length) and a different SESAM (Batop GmbH SAM-1550-10-5ps) are utilized. For the frequency stabilization, a piezoelectric transducer (PZT) is integrated with the bare gain fiber. Other items are the same as those of the 21-GHz fiber laser. To secure a better phase noise performance, the output of the 4.6-GHz fiber laser is divided into two paths by a 50/50 optical coupler (OC), and one path serves as the output. The other path is detected and converted into the electric signal by a high-speed PD. The second-harmonic frequency of the electric signal, i.e., at ~9.2 GHz, is firstly filtered by an electric BPF (Mini-Circuits ZVBP-10R5G+) and amplified after an electric amplifier (EA, Mini-Circuits ZVA-183-S+). Then, the harmonic signal is compared to a stable reference signal from a microwave signal generator (MSG, Keysight 83711A) in a mixer (Mini-Circuits ZX05-153+). The phase error signal from the mixer is filtered by a lowpass filter (LPF, Mini-Circuits SLP-1.9+) and processed by a proportional-integral-derivative controller (PID, SRS SIM960) that generates the correct controlled signal. This controlled signal is applied to the intracavity PZT to rapidly control the length of the GHz fiber laser cavity by stretching the bare gain fiber for stabilizing the fundamental repetition rate.

Figure S11 | Configuration of a frequency-stabilized mode-locked fiber laser with a fundamental repetition rate of 4.6 GHz. Left inset shows the photo of the GHz fiber laser cavity. SESAM, semiconductor saturable absorber mirror. EYDF, Er^3+^/Yb^3+^ co-doped phosphate fiber. PZT, piezoelectric transducer. DF, dielectric film. TC, temperature controller. PC, polarization controller. WDM, wavelength division multiplexer. SM-LD, single-mode laser diode. ISO, isolator. OC, optical coupler. PD, photodetector. BPF, bandpass filter. EA, electric amplifier. MSG, microwave signal generator. LPF, lowpass filter. PID, proportional-integral-derivative controller.

We characterize the basic performance of the frequency-stabilized mode-locked GHz fiber laser, as shown in **Fig. S12**. **Fig. S12a** illustrates the output power as a function of the pump power. As the pump power increases, the GHz fiber laser operates from RSQSML to CWML when the pump power is over 85 mW. The inset of **Fig. S12a** shows the oscilloscopic trace of the CWML pulses, and the corresponding optical spectrum is shown in **Fig. S12b**, which is centered at 1567 nm with a 3-dB bandwidth of 2 nm. The closeup of the optical spectrum identifies a longitudinal mode spacing of 0.04 nm, corresponding to a fundamental repetition rate of ~4.6 GHz. **Fig. S12c** illustrates the RF spectrum with a RBW of 1 kHz, which also identifies a fundamental repetition rate of ~4.6 GHz. The stability of output power over 500 s under the repetition-rate stabilization is measured and depicted in **Fig. S12d**, which shows a relative standard deviation of 0.034%. **Fig. S13** illustrates the phase noise and integrated timing jitter of the frequency-stabilized mode-locked GHz fiber laser. It shows that the phase noise is reduced at the frequency range below 1 kHz. The integrated timing jitter is reduced from 17 ps to 598 fs at a frequency range from 10 Hz to 1 kHz, corresponding to a reduction factor of 28.5.

Figure S12 | Basic performance of the frequency-stabilized mode-locked fiber laser with a fundamental repetition rate of 4.6 GHz. a. Output power as a function of the pump power. Inset shows the oscilloscopic trace of the CWML pulses with time spans of 4 ns (top) and 10 μs (bottom). b. Optical spectrum. The inset depicts the closeup of the optical spectrum that clearly manifests that longitudinal mode spacing of ~0.04 nm. c. RF spectrum measured with a RBW of 1 kHz. Inset shows the RF spectrum over a wider span ranging from 0 to 15 GHz. d. Power evolution over 500 s. RSD, relative standard deviation.

Figure S13 | Phase noise (a) and integrated timing jitter (b) of the frequency-stabilized mode-locked fiber laser with a fundamental repetition rate of 4.6 GHz.

Supplementary Reference

1. Chen, H.-W., et al. 3 GHz, fundamentally mode-locked, femtosecond Yb-fiber laser. *Optics Letters* **37**, 3522 (2012).

2. Cheng, H. H. et al. 5 GHz fundamental repetition rate, wavelength tunable, all-fiber passively mode-locked Yb-fiber laser. *Optics Express* **25**, 27646 (2017).

3. Cheng, H. H. et al. High-repetition-rate ultrafast fiber lasers. *Optics Express* **26**, 16411 (2018).

4. Wang, W. L. et al. Gain-guided soliton: Scaling repetition rate of passively modelocked Yb-doped fiber lasers to 12.5 GHz. *Optics Express* **27**, 10438 (2019).

5. Byun, H. et al. Compact, stable 1 GHz femtosecond Er-doped fiber lasers. *Applied Optics* **49**, 5577 (2010).

6. Chen, J. et al. Fundamentally Mode-locked 3 GHz Femtosecond Erbium Fiber Laser. in *Ultrafast Phenomena XVI* (eds. Corkum, P., Silvestri, S., Nelson, K. A., Riedle, E. & Schoenlein, R. W.) **92**, 732–734 (Springer Berlin Heidelberg, 2009).

7. Song, J. Z. et al. All-polarization-maintaining, semiconductor saturable absorbing mirror mode-locked femtosecond Er-doped fiber laser with a gigahertz fundamental repetition rate. *Laser Physics Letters* **16**, 095102 (2019).

8. Song, J. Z. et al. Compact low-noise passively mode-locked Er-doped femtosecond all-fiber laser with 2.68 GHz fundamental repetition rate. *Applied Optics* **58**, 1733 (2019).

9. Muñoz-Marco, H. et al. Theoretical and experimental comprehensive study of GHz-range passively mode-locked fiber lasers. *Applied Optics* **59**, 6817 (2020).

10. Gao, X. B. et al. Stable 5-GHz fundamental repetition rate passively SESAM mode-locked Er-doped silica fiber lasers. *Optics Express* **29**, 9021 (2021).

11. McFerran, J. J. et al. A passively mode-locked fiber laser at 1.54 μm with a fundamental repetition frequency reaching 2 GHz. *Optics Express* **15**, 13155 (2007).

12. Martinez, A. & Yamashita, S. Multi-gigahertz repetition rate passively modelocked fiber lasers using carbon nanotubes. *Optics Express* **19**, 6155 (2011).

13. Thapa, R. et al. A. All-fiber fundamentally mode-locked 12 GHz laser oscillator based on an Er/Yb-doped phosphate glass fiber. *Optics Letters* **39**, 1418 (2014).

14. Zhou, Y. et al. Composite filtering effect in a SESAM mode-locked fiber laser with a 3.2-GHz fundamental repetition rate: switchable states from single soliton to pulse bunch. *Optics Express* **26**, 10842 (2018).

15. Chen, X. W. et al. High-power femtosecond all-fiber laser system at 1.5 µm with a fundamental repetition rate of 4.9 GHz. *Optics Letters* **46**, 1872 (2021).

16. Lin, W. et al. Manipulating the polarization dynamics in a >10-GHz Er^3+^/Yb^3+^ fiber Fabry-Pérot laser. *Optics Express* **30**, 32791 (2022).

17. Qin, C. Y. et al. Electrically controllable laser frequency combs in graphene-fibre microresonators. *Light Science & Applications* **9**, 185 (2020).

18. Cheng, H. H. et al. Theoretical and experimental analysis of instability of continuous wave mode locking: Towards high fundamental repetition rate in Tm^3+^-doped fiber lasers. *Optics Express* **24**, 29882 (2016).

19. Zeng, J. J., Akosman, A. E. & Sander, M. Y. Scaling the repetition rate of thulium-doped ultrafast soliton fiber lasers to the GHz regime. *Optics Express* **26**, 24687 (2018).

20. Tang, G. W. et al. 4.3 GHz fundamental repetition rate passively mode-locked fiber laser using a silicate-clad heavily Tm^3+^-doped germanate core multimaterial fiber. *Optics Letters* **47**, 682 (2022).

21. Liang, Z. H. et al. >10 GHz femtosecond fiber laser system at 2.0 μm. *Optics Letters* **47**, 1867 (2022).

22. Pottiez, O. et al. Gain-driven spectral-temporal noise-like pulse dynamics in a passively mode-locked fiber laser. *Optics Express* **27**, 34742 (2019).

23. Amrani, F. et al. Passive harmonic mode locking of soliton crystals. *Optics Letters* **36**, 4239 (2011).

24. Haus, H. A. Parameter ranges for CW passive mode locking. *IEEE Journal of Quantum Electronics* **12**, 169–176 (1976).

25. Keller, U. et al. Semiconductor saturable absorber mirrors (SESAM’s) for femtosecond to nanosecond pulse generation in solid-state lasers. *IEEE Journal of Selected Topics in Quantum Electronics* **2**, 435–453 (1996).

26. Schibli, T. R. et. al. Suppression of Q-switched mode locking and break-up into multiple pulses by inverse saturable absorption. *Applied Physics B* **70**, S41–S49 (2000).

27. Keller, U. Recent developments in compact ultrafast lasers. *Nature* **424**, 831–838 (2003).

28. Haboucha, A. et al. Analysis of soliton pattern formation in passively mode-locked fiber lasers. *Physical Review A* **78**, 043806 (2008).

29. Hönninger, C. et al. Q-switching stability limits of continuous-wave passive mode locking. *Journal of the Optical Society America B* **16**, 46 (1999).

30. Yu, M. J. et al. Breather soliton dynamics in microresonators. *Nature Communications* **8**, 14569 (2017).

31. Peng, J. S. et al. Breathing dissipative solitons in mode-locked fiber lasers. *Science Advances* **5**, eaax1110 (2019).

32. Dudley, J. M. et al. Instabilities, breathers and rogue waves in optics. *Nature Photonics* **8**, 755–764 (2014).
